# Novel Polymorphic Patterns for Elacestrant Dihydrochloride

**DOI:** 10.3390/pharmaceutics17060745

**Published:** 2025-06-05

**Authors:** Zia Uddin Masum, P. Grant Spoors, Matt D. Burke, Vivek Gupta

**Affiliations:** 1 Department of Pharmaceutical Sciences, College of Pharmacy and Health Sciences, St. John’s University, 8000 Utopia Parkway, Queens, NY 11439, USA; 2 Stemline Therapeutics, Inc., A Menarini Group Company, 750 Lexington Avenue, 4th Floor, New York, NY 10022, USA; grantspoors@gmail.com

**Keywords:** polymorphism, elacestrant, characterization, amorphous, crystalline, anti-solvent addition, polymer templating, solvent-drop grinding

## Abstract

**Objective:** This study expands on the polymorphic characterization of elacestrant dihydrochloride, developed by Stemline Therapeutics and approved by the FDA in 2023. The article focuses on more extensive polymorphism screening using various methods and solvents to discover the new polymorphism forms of this molecule, besides identifying three polymorphic forms in the previously published studies. **Methods:** The crystalline and amorphous elacestrant hydrochloride solubility was assessed, and crystals were formed, followed by polymorph screening using 40 non-conventional solvents via different techniques to obtain the new polymorphic forms. XRPD, NMR, DSC, TGA, IC, and HPLC were used for solid-state characterization. **Results:** Patterns A, B, C, D, E, F, and G, and previously published forms 1,3, were identified in multiple studies during the extensive polymorphism screening using various methods and numerous solvent systems. Solid state characterization and purity analysis were completed using different relevant instruments. After the characterization, it was found that Pattern A was the most stable, like the desired/most stable Form 1, but it had fewer crystals; Pattern B is like Form 3 but a unique XRPD pattern; Pattern D is degradant; Pattern C, E, F, and G are considered as the new pattern of elacestrant along with patterns A and B. **Conclusions:** With XRPD, six new patterns (A, B, C, E, F, G) were identified. Patterns A, C, and E are promising crystalline candidates for further analysis and scale-up.

## 1. Introduction

Cancer is a leading cause of death globally. According to the American Cancer Society, 20 million new cases and 9.7 million deaths were recorded worldwide in 2022. By 2050, the number of cancer cases is estimated to rise to around 35 million worldwide [1,2]. In the USA, 2 million new cancer cases will be diagnosed, and 0.6 million people will die from the disease in 2024. The most common cancers include cancers of the breast, lung, prostate, colon, skin, pancreatic, blood, bladder, kidney, liver, etc. [3], with the most common cancer and the leading cause of death among women being breast cancer [4].

Breast cancer is the second leading cause of cancer deaths (15–16%) in women after lung cancer [5,6]. According to WHO, approximately 2.3 million women were diagnosed with breast cancer in 2022, resulting in 0.67 million deaths globally [7]. In the USA, there are an estimated 310,720 new cases of invasive breast cancer along with 56,500 new cases of non-invasive (in situ) breast cancer in women in 2024. Additionally, an estimated 42,250 U.S. women will die from breast cancer [8]. Breast cancer not only affects women but also all individuals, regardless of gender. About 2790 new cases of invasive breast cancer will be diagnosed in men, with approximately 530 male deaths (1 in 726) in the USA in 2024 [9,10].

About 80% of breast cancers are estrogen receptor (ER)-positive. Endocrine therapy, the primary treatment for ER-positive cancers, includes aromatase inhibitors and selective estrogen receptor modulators, like tamoxifen, to inhibit estrogen-driven tumor growth [11]. However, the effectiveness of endocrine therapy is often limited due to the development of resistance, reducing the effectiveness of the treatments [12].

A newer class of endocrine therapy, a selective estrogen receptor degrader (SERD), targets and explicitly reduces ER activity [13]. Due to limited oral bioavailability, the FDA approved Fulvestrant as the first SERD to be administered as an intramuscular injection in 2002 [14,15,16,17].

Elacestrant is the first orally available selective estrogen receptor degrader (SERD) (Orserdu^®^), developed by Stemline Therapeutics Inc, (New York, NY, USA) Elacestrant received FDA approval in January 2023 for ER-positive, HER2-negative advanced breast cancer with ESR1 mutation [18,19,20]. Elacestrant is available in oral dosage forms of 86 mg and 345 mg tablets [14,21]. Elacestrant dihydrochloride is the active ingredient of the drug Product [22].

Elacestrant dihydrochloride (IUPAC name (6R)-6-(2-(N-(4-(2-(ethylamino)ethyl)benzyl)-N-ethylamino)-4-methoxyphenyl)-5,6,7,8 tetrahydronaphthalen-2-ol dihydrochloride) [23] is the salt form of the elacestrant, freely soluble in 0.01 N HCL and soluble in water (25 mg/mL). It is a white to off-white to grey solid powder [23,24]. The molecular properties of elacestrant dihydrochloride are outlined below in Table 1 [25,26,27].

Although elacestrant oral administration is simple and convenient for patients, systemic drug absorption, followed by dissolution, can depend on the polymorphic form of the API. The selected molecule is in its salt form, which does not significantly impact dissolution. However, the stability of these molecules remains crucial. Selecting the lowest energy crystalline polymorph during drug development is important, as higher energy polymorphic forms may have serious pharmacokinetic consequences, like a very prominent case of ritonavir [28]. Our previous studies demonstrated that elacestrant dihydrochloride converted to another polymorphic form under varying humidity and temperature conditions, which is a very unstable polymorphic form. This study continues the search for new possible polymorphic forms of the drug to find the stable polymorphic form [24]. Additionally, understanding the polymorph landscape for a molecule helps ensure the most appropriate polymorphic form for further clinical development and commercialization, underscoring the importance of tailored drug development and formulation strategies [29].

In addition, the need to assess polymorphism in the pharmaceutical industry primarily arises for two reasons. Firstly, polymorphism is inevitable and may occur during discovery, development, and manufacturing processes as an inherent characteristic of materials. Secondly, the formulator may modify the physicochemical properties of a given compound by exploiting different polymorphs [30]. McCrone stated in 1965, “Every compound has different polymorphic forms and that, in general, the number of forms known for that compound is proportional to the time and money spent researching it” [31].

Modifying the physical forms of a compound, such as polymorphs, solvates, amorphous structures, salts, co-crystals, and hydrates, is a common strategy for enhancing and optimizing drug characteristics [29]. Solubility, dissolution, bioavailability, and physical/chemical stability are critical in drug discovery and development. For example, Chloramphenicol palmitate polymorphs show significantly different bioavailability in human studies [32].

Extensive studies were performed to assess the polymorphic behavior of elacestrant dihydrochloride. Our recently published studies and publicly available patent application for elacestrant (filed by Radius Pharmaceuticals, from which Menarini Group bought the commercial rights) described the discovery and characterization of three polymorphic forms of elacestrant, identified as Form 1, Form 2, and Form 3. These forms are referred to as such throughout the manuscript [24,33,34].

Generating polymorphic forms involves various methods, including crystallization from solvents, thermal activation of solid substrates, crystallization from the melt, crystallization in nano-confined structures, desolvation/dehydration, seeding/pseudo seeding, solution-mediated polymorphic transformation, solid-state polymorphic transformation, mechanical activation, directed crystallization on molecular substrates, exposure to vapor, and crystallization with additives. Solvent-dependent polymorphism is also a common technique, such as arbidol’s conformational preferences in deuterated chloroform and dimethyl sulfoxide at 25 °C, using 2D NOESY, which potentially impacts pharmaceutical activity. Advanced methods include laser-induced crystallization, supercritical fluid crystallization, and structure prediction [31,35]. Crystallization is a widely used approach, like solvent drop grinding, polymer template crystallization, and crystallization by anti-solvent addition, all of which are very prominent techniques for facilitating the formation of co-crystals and crystallization [36,37,38]. For this study, extensive screening was performed using non-conventional methods to discover novel polymorphs of elacestrant and supplement the three forms mentioned in the earlier published study [24]; amorphous and crystalline forms of elacestrant were utilized in this study. This comprehensive approach enhances the understanding of polymorphic behavior and expands the potential forms of a given compound. This is one of the only polymorphic pattern studies describing the discovery of new polymorphic forms of elacestrant. The additional polymorphic forms identified in this paper are designated by an alphabetical letter scheme to differentiate them from the previously identified polymorphs, i.e., Form 1, 2, and 3 [24]. This study used the term “Polymorphic Pattern” to define the new polymorphic form, incorporating both the anhydrous form (sole polymorphism) and the hydrate form of the molecule, which aligns with the FDA’s solid polymorphism guidance [39].

## 2. Materials and Methods

### 2.1. Materials

Elacestrant Form 2/3 (anhydrous/hydrate mixture that exhibits lower stability and is a dynamic mixture of anhydrous, which is Form 2, and hydrated states, which is Form 3 influenced by ambient RH) was used as the input material for these studies. The API was provided by Radius Health/Stemline Therapeutics, Inc., New York, NY, USA. All organic solvents and other excipients were purchased from scientific vendors. The studies were performed by Pharmorphix, Inc. (Cambridge, MA, USA) under the scientific guidance of Radius Health/Stemline Therapeutics, Inc. (New York, NY, USA).

### 2.2. Instruments and Method

#### 2.2.1. X-Ray Powder Diffraction (XRPD)

Bruker AXS C2 GADDS diffractometer used for XRPD (Bruker AXS, Madison, WI, USA) using Cu Kα radiation (40 kV, 40 mA). Its automated XYZ stage contains a laser video microscope that helps fix the auto-sample positioning and a HiStar 2-dimensional area detector. A Göbel multilayer mirror (single) coupled with a pinhole collimator (0.3 mm) consists of the X-ray optics.

The beam divergence, i.e., the adequate size of the X-ray beam on the sample, was approximately 4 mm. A θ-θ continuous scan mode was employed with a detector distance of 20 cm, which gives an effective 2θ range of 1.5–32.5°. Typically, the sample is exposed to the X-ray beam for 120 s. Diffrac Plus EVA v15.0.0.0 software is used for data analysis and presentation. Samples were run under ambient conditions and prepared as flat plate specimens (whole discs) using a mechanical disc press. Alongside this diffractometer, several equivalent diffractometers were utilized for comprehensive analysis throughout the studies, as listed in the Supplementary Information (Supplementary Section S2.2.1).

#### 2.2.2. Single-Crystal X-Ray Diffraction (SCXRD)

Data were generated using a Rigaku Oxford Diffraction Supernova Dual Source diffractometer with Cu at zero and an Atlas CCD detector with an Oxford Cryosystems Cobra cooling device. The radiation used was of Cu Kα or Mo Kα. Bruker AXS SHELXTL suite or OLEX crystallographic software was used to solve and refine structures [40]. Unless stated otherwise, hydrogen atoms attached to carbon were placed geometrically and refined with a riding isotropic displacement parameter. Hydrogen atoms attached to heteroatoms were located in a different Fourier synthesis and refined freely with an isotropic displacement parameter. Mercury is used as a reference diffractogram for crystal structure generation.

#### 2.2.3. Nuclear Magnetic Resonance (NMR)

^1^H NMR spectra were collected on a Bruker 400 MHz instrument (Bruker, Madison, WI, USA) equipped with an auto-sampler and controlled by a DRX400 console (Bruker). Samples were prepared in DMSO-d6 solvent unless otherwise stated. Automated experiments were acquired using ICON-NMR configuration within Topspin software (version 1.3), using standard Bruker-loaded experiments (^1^H, ^13^C {^1^H}, DEPT135). Off-line analysis was performed using ACD Spectrus Processor.

#### 2.2.4. Differential Scanning Calorimetry (DSC)

TA Instruments Q2000 was used to collect data (TA Instruments, New Castle, DE, USA) equipped with a 50-position auto-sampler. Typically, 1–5 mg of each sample was placed in a pin-holed aluminum pan, and a 10 °C/min rate of heat was maintained from 25 °C to 300 °C. Modulated DSC temperature was measured using an underlying heating rate of 2 °C/min and temperature modulation parameters of ±0.636 °C (amplitude) every 60 s (period). Universal Analysis software (version 4.5A), was used to analyze data. Along with this instrument, another DSC was used to analyze these studies. Please refer to the Supplementary Information (Supplementary Section S2.2.2).

#### 2.2.5. Thermo-Gravimetric Analysis (TGA)

A TA Q500 TGA Instrument was used to collect data (TA Instruments, New Castle, DE, USA), equipped with an auto-sampler containing 16-position. Usually, each sample of 5–10 mg was placed onto a DSC pan of a pre-tared aluminum, and a 10 °C/min heat rate was maintained from room temperature to 350 °C. The 60 mL/min heat rate was kept constant throughout the sample. The data were analyzed using Universal Analysis software. Another TGA was required to analyze these experiments; please refer to the Supplementary Materials (Supplementary Section S2.2.3).

#### 2.2.6. Chemical Purity Determination by HPLC

An Agilent HP1100 series HPLC was used to analyze the purity of API (Agilent, Waldbronn, Karlsruhe, Germany) fitted with a Diode Array Detector (DAD), and ChemStation software (version B.04.03) was used for this. The full method details are provided mentioned below as Table 2.

#### 2.2.7. Ion Chromatography (IC)

A Metrohm 930 Compact IC Flex attached with an autosampler (858 Professional), Herisau, Switzerland and an 800 Dosimo dosage unit monitor was used for the data collection, and IC MagicNet software (version 3.1) was used. Samples were prepared by accurately weighing (stock solutions), completely dissolving them, and diluting them appropriately before testing. Quantification was achieved by comparing the results with standard solutions of analyzed known ion concentrations. IC method for anion chromatography is given below as Table 3.

### 2.3. Sample Preparation for Single-Crystal X-Ray Diffraction (SCXRD)

Firstly, crystalline formation ability was checked by using the input material of the Elacestrant. An amount of 10 mg of the starting/input material of elacestrant was treated with increasing aliquots of one of 14 selected solvent systems until complete dissolution was observed or a maximum of 100 vol. had been added. The solubility assessment was performed at 40 °C. Clear solutions and partially soluble suspensions were cooled to 5 °C at 0.1 °C/min and maintained at 5 °C for two days. Solutions that had frozen were allowed to warm to room temperature. After cooling, clear solutions were warmed to room temperature and slowly evaporated, while solutions containing crystals were left at room temperature to encourage Ostwald ripening.

Suspensions were subjected to heating/cooling cycles from 25 °C to 50 °C (4 h at each temperature) for two days and were then extracted at 50 °C, filtered, and split into three parts. One-third was allowed to evaporate slowly in ambient conditions, another was cooled to 5 °C, and the final third was kept for further studies.

### 2.4. Preparation of Amorphous Material

Amorphous material was generated from the elacestrant (Form 2/3) by lyophilization from t-BuOH/H_2_O (1:1). Elacestrant (Form 2/3, 800 mg) was weighed in duplicate into 20 mL scintillation vials and dissolved, whilst stirring at 50 °C, in t-BuOH/H_2_O (20 vol., 16 mL). The solution was filtered and decanted into HPLC vials (700 μL, ~35 mg of elacestrant per vial). The vials were snap-frozen in a dry ice/acetone bath and lyophilized overnight.

### 2.5. Crystallization Methodologies and Polymorph Screening

The choice of crystallization method significantly influences which polymorphic form is produced, and it is essential to perform crystallization using various techniques and conditions when looking for polymorphs [29,41]. Classical crystallization methods used in this study are given below with each process [42].

#### 2.5.1. Solvent-Mediated Techniques

These are classical techniques used for generating crystalline material. Theoretically, crystallization occurs when the concentration of a compound in a solvent is higher than its solubility product. Generally, crystallization is kinetically hindered, and crystals grow only under supersaturated solutions.

For a crystallization screen, solvents with highly diverse properties should be chosen (hydrogen bond donor/acceptor propensity, dipole moment, dielectric constant, viscosity, etc.). Often, solvent mixtures help obtain systems with suitable solubilities, polarities, etc. It also must be ensured that the substance is chemically stable in the given solvents or solvent mixtures.

##### Maturation/Slurry Ripening

Mature experiments (or slurry ripening) are often performed in various solvents or solvent mixtures and subjected to heat–cool cycles to investigate crystalline forms. Repeated heating and cooling cycles may increase the degree of crystallinity or convert a meta-stable state (or out-of-equilibrium state of amorphous material) into a more thermodynamically stable crystalline form. The conversion rate and extent depend upon the input material’s solubility [43].

For thermodynamic reasons, the system can only evolve towards more stable forms. Therefore, obtaining a less stable crystalline phase is impossible if the starting material is crystalline. A greater number of patterns may be formed if the starting material is amorphous.

##### Procedure Using Maturation Chamber

Suspensions for maturation were placed in a platform shaker incubator (Heidolph Titramax/Incubator 1000), Illinois, USA and subjected to a series of heat–cool cycles from ambient to approximately 50 °C. This is achieved by switching the heating on or off every 4 h. Shaking is maintained throughout the process. The maturation chamber, along with the temperature profile, is provided below.

##### Procedure Using Polar Bear Device

Suspensions were stirred (400–600 rpm) in a Polar Bear (Cambridge Reactor Design) Cambridge, UK for four hours at 40 °C. The samples were then cooled to 25 °C and stirred for four hours. The cycle was then repeated.

##### Cooling Crystallization

Crystallization can be obtained by lowering the temperature of a clear solution. The solubility of most materials decreases with decreasing temperature, so cooling can be used to generate supersaturation. In many cases, however, the solubility of a material remains high even at low temperatures, or the solubility changes very little over the temperature range of interest. In these cases, other methods for the creation of supersaturation must be considered (such as solvent evaporation) [39].

Solutions were cooled to 5 °C at 0.1 °C/min in a Polar Bear and stirred at this temperature for 24 h. All solids were filtered and analyzed ‘damp’ by XRPD and subsequently allowed to dry under ambient conditions for 1–4 days and re-analyzed ‘dry’ by XRPD. Any remaining solutions were evaporated.

##### Controlled Evaporation

**Scheme 1:**
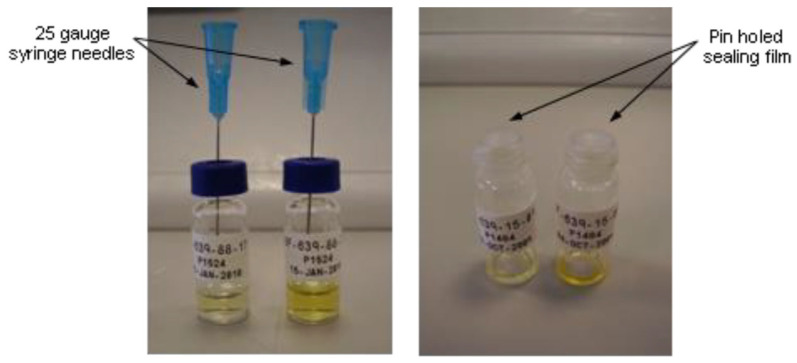
Slow evaporation experiments.

Crystallization can be generated by controlled evaporation of a clear, particulate-free solution. This is especially true when the solvent has relatively high vapor pressure. At approximately constant temperature, the solvent is removed from the system, thereby increasing the solute concentration. The crystal nucleation and growth are obtained when some maximum supersaturation is reached [44]. This technique also has the advantage that since the samples are slowly evaporated mentioned as Scheme 1, it is often possible to generate large single crystals suitable for SCXRD.

Solutions were evaporated at ambient conditions by inserting a needle into the septum cap of the vials. The samples were allowed to slowly evaporate to dryness or until a solid appeared at ambient conditions.

Crystallization formations were prepared for the crystalline and amorphous samples using the above technique.

#### 2.5.2. Solubility Assessment, Crystallization, and Polymorph Screening Procedure (Crystalline API)

Elacestrant (Form 2/3, 30 mg) was weighed into HPLC vials, and a stirrer bar was added. The samples were treated with solvent (10 vol., 300 μL) whilst stirring at 25 °C, 500 rpm, and visual assessments were performed after 10 min.

Samples that remained as suspensions were treated with additional solvent until they dissolved or a maximum of 50 vol. was added. The temperature then increased to 40 °C, and visual assessments were performed after 10 min. Solutions obtained were cooled from 50 to 5 °C at 0.1 °C/min and held for 24 h. Samples that remained suspensions were matured in heat–cool cycles between 50 °C and RT (8 h/cycle) in a maturation chamber for 2 days and filtered under suction for 1 h.

The solids were analyzed by XRPD after isolation and were denoted ‘damp’. After 4 days of air-drying at ambient, the samples were re-analyzed and were denoted ‘dry’. Samples which remained as solutions were left to evaporate through a needle inserted into the septum of the vial

#### 2.5.3. Polymer Template Crystallizations (Crystalline API)

Elacestrant (Form 2/3, 50 mg) was weighed into HPLC vials whilst stirring at 40 °C, 600 rpm on a Polar Bear device. Samples were dissolved in the minimum volume of solvent (ethanol/water 96:4, 30 vol., or MeOH, 10 vol.). Low solubility was observed in chloroform (50 vol.). Therefore, the resulting suspension was filtered, and the filtrate was used to seed with the polymer. An amount of 5% wt. (2.5 mg) of the polymer was added to each solution. The samples were held at 40 °C for 15 min, and then the sample was allowed to evaporate to dryness at RT with the vial cap removed. The residue obtained was analyzed by XRPD. The solubility of the polymers differed in each solvent and often did not completely dissolve in solution.

#### 2.5.4. Solvent-Drop Grinding Experiments (Crystalline API)

Elacestrant (Form 2/3, 30 mg) was weighed into HPLC vials with two stainless steel ball bearings (3 mm ø), and a drop of dry solvent (15 μL) was added. The samples were ground in a Fritsch planetary mill for 2 h (500 rpm). The resulting solids were analyzed by XRPD.

#### 2.5.5. Solubility Assessment and Polymorph Screening Procedure (Amorphous API)

Amorphous solid (~35 mg) was treated with an aliquot of solvent (10 vol., 300 μL) whilst stirring at 25 °C, 500 rpm. Visual assessments were made after 10 min. Samples that remained as suspensions were treated with additional solvent (up to a maximum of 50 vol.) until dissolution occurred. The temperature was then increased to 40 °C, and visual assessments were made after 10 min.

Samples that formed the solution were cooled from 40 to 5 °C at 0.1 °C/min and held for 24 h. Any solutions remaining after this time were allowed to evaporate slowly at RT to produce single crystals. These experiments were repeated with the addition of 2.5 vol. solvent and stirring at 40 °C. Samples that remained suspensions were matured in heat–cool cycles between 40 °C and 25 °C (8 h/cycle) for 2 days on a Polar Bear device.

All suspensions obtained from cooling/maturation were filtered under suction for 1 h, and the resulting solids were analyzed by XRPD, denoted as ‘damp’ samples. After 4 days of drying under ambient conditions to air-dry, samples were re-analyzed by XRPD and denoted as ‘dry’ samples.

#### 2.5.6. Crystallization by Anti-Solvent Addition (Crystalline API)

Anti-solvent crystallization (or drown-out crystallization) is commonly used to precipitate material from a solution. Adding a miscible antisolvent to a solute solution reduces the original solubility of the solute, increasing the supersaturation and thus causing precipitation. The selected anti-solvent should be miscible with the solvent at any proportion, and the solute should be relatively insoluble.

Solutions were treated with anti-solvent in aliquots at 50 °C until it became cloudy or a precipitate formed. Any precipitates were filtered, and XRPD analyzed the aliquot. All samples were cooled to 5 °C in the fridge and held isothermally. Suspensions at 5 °C were filtered and analyzed ‘damp’ by XRPD and subsequently allowed to dry under ambient conditions for 24 h.

Elacestrant dihydrochloride (Form 2/3, 50 mg) was weighed into HPLC vials and dissolved in the minimum volume of solvent (ethanol/water 96:4, 20–30 vol., or methanol, 5 vol.) whilst stirring and heating at 50 °C, 600 rpm on a Polar Bear device.

Once dissolved, anti-solvent was added slowly until a 1:1 ratio of antisolvent/solvent (*v*/*v*) was reached. A stirring for 5–10 min observations were recorded. The ethanol/water sample solutions were transferred to scintillation vials prior to anti-solvent addition. Additional anti-solvent (up to a maximum ratio of 5:1 antisolvent: solvent) was added until precipitation was observed.

For thick suspensions, aliquots were taken at 50 °C, filtered on a Millipore plate, and analyzed by XRPD. All samples were then cooled to 5 °C in the fridge for 16 h. The material was initially analyzed as ‘damp’ by allowing the solvent to filter through each well under gravity. Then, each well was re-analyzed as ‘dry’ after allowing at least 24 h for air drying at RT.

## 3. Results and Discussion

### 3.1. Input/Starting API Characterization, Elacestrant (Form 2/3)

**Figure 1:**
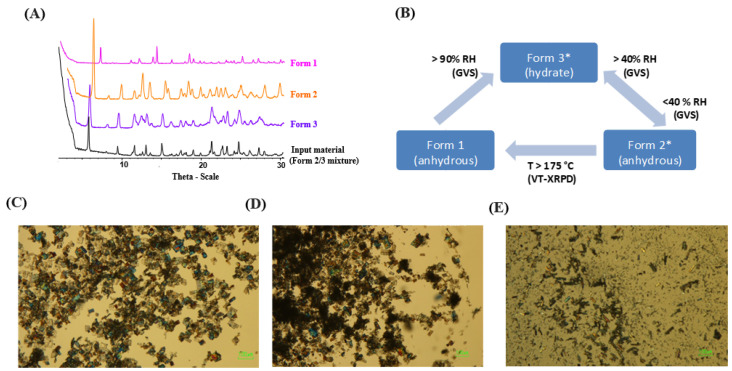
Elacestrant dihydrochloride: (**A**) XRPD of Form 2/3 and Forms 1, 2, and 3; (**B**) conversion of Forms 1, 2, and 3 (* undesired form); (**C**–**E**) Polarised Light Microscopy (PLM) image of Form 1, Form 2/3, Form 3. Scale bar: 100 μm

An overlay of the XRPD diffractograms for the elacestrant was consistent with a mixture of Form 2 and Form 3, Form 1, Form 2, as well as Form 3, as shown in Figure 1A. Figure 1B demonstrates the interconversion between Form 1, Form 2, and Form 3 under varying humidity and temperature conditions. Specifically, it was observed that Form 2 transitions to Form 3 at humidity levels exceeding 40% relative humidity (RH). Here, it was found that Forms 2 and 3 are more unstable under typical room humidity, which makes them undesirable polymorphic forms. We used Form 2 and Form 3 mixture for our research as a starting material to consider undesirable polymorphic forms. In brief, Figure 1 C–E provides crystal images obtained through Polarized Light Microscopy (PLM) of the desired Form 1, the undesired form mixture of Forms 2 and 3, and the hydrated Form 3 to understand the crystal nature of the molecules [24]. Elacestrant (Form 2/3) exhibited good chemical purity (99.2% by HPLC). The ^1^H NMR spectrum was consistent (Figure S1A), and as seen in Figure S1B, thermal analysis of the material showed similar events as the previously studied batch for Form 2/3, a broad endotherm from RT to 150 °C and an endothermic event at ~160 °C (onset). However, the weight loss associated with the broad endotherm (RT to 150 °C) was greater for this batch (8.4% wt. vs. 5.2% wt.), this equates to approximately 2.5 equivalents of water, as compared to our previous Form 2/3 API lot [24]. The onset of degradation occurred earlier (~200 °C), so it was impossible to observe a recrystallization–melt (exothermic–endothermic) event in this batch [24].

### 3.2. Single-Crystal Experiments

**Figure 2:**
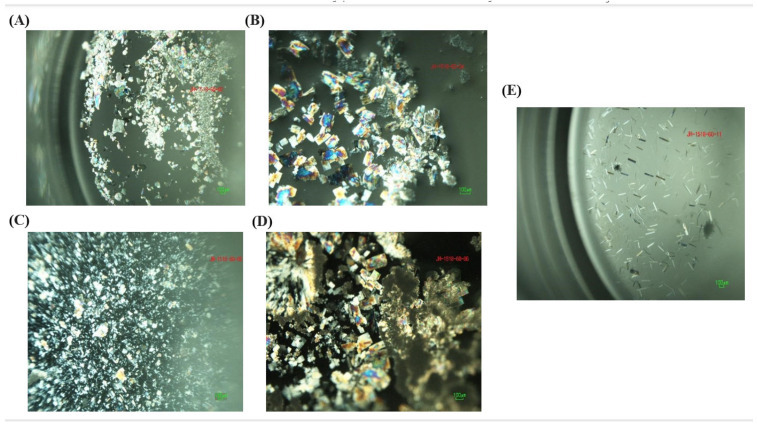
(**A**) Cooling in methanol with storage at 5 °C; (**B**) cooling in water–ethanol (1:99) with storage at 5 °C; (**C**) cooling in water–ethanol (2:98) with storage; (**D**) cooling in water–ethanol (3:97) with storage at 5 °C; (**E**) cooling in water with storage at 5 °C.

**Figure 3:**
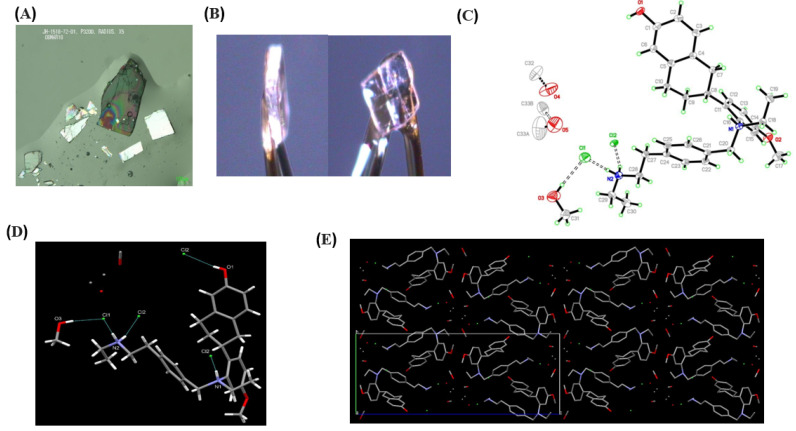
(**A**) Optical micrograph of the crystalline sample; (**B**) optical micrograph of the crystal selected; (**C**) molecular configuration of elacestrant methanol solvate (ORTEP); (**D**) hydrogen bonding within the elacestrant methanol solvate structure (white = H2, red = O3, blue = N2, green = Cl2 indicated the respective molecule); (**E**) crystal packing of elacestrant methanol solvate viewed down the a-axis (red = O3, blue = N2, green = Cl2 indicated the respective molecule).

From the evaluation of the samples, a range of cubic crystals, plates, and needles/rods were obtained from methanol, ethanol, water, and ethanol–water mixtures, as shown in Figure 2 and Table S1. Samples from methanol were submitted for screening, and a single-crystal X-ray diffraction study was carried out on the sample of methanol solvate. Methanol solvents produced cubic crystals, as shown in Figure 3A,B.

As shown in Figure 3C, the asymmetric unit contains one fully ordered molecule of elacestrant (which has been protonated at N1 and N2), two chloride ions, one ordered molecule of methanol, and two further partially occupied and disordered methanol solvent (O4 and O5). Anisotropic atomic displacement ellipsoids for the nonhydrogen atoms are shown at the 50% probability level. Hydrogen atoms are displayed with an arbitrarily small radius.

As Figure 3D shows, hydrogen bonding is present within the crystal lattice with strong charge-assisted hydrogen bonds between N1 and Cl2 and N2 with Cl1 and Cl2. There are also additional O-H···Cl hydrogen bonds between the alcohol groups (elacestrant and the ordered methanol solvent) and the chloride ions. Details of hydrogen bonding interactions are given below as Table 4.

Figure 3E shows a view of the crystal packing of the elacestrant methanol solvate, looking approximately down the crystallographic a-axis showing a hydrogen-bonded network of elacestrant^2+^ and Cl^−^ ions with channels of disordered methanol solvent. The molecules within these channels are disordered, making assignment difficult, and methanol may be present. For clarity, all hydrogen atoms have been removed.

A summary of all sample details and crystal data for the elacestrant methanol solvate is provided in Table 5.

Here, elacestrant methanol solvate crystallizes in the orthorhombic system, space group *P*2_1_2_1_2_1_, with the final R1 [I > 2α(I)] = 4.12%. Additionally, data, collection, and structure refinement for elacestrant methanol solvate can be found in Table S2.

Figures S2 and S3 showed the pattern of the elacestrant methanol solvate (in black), which is very similar to the experimental XRPD pattern of elacestrant Form 3 (green), a hydrated form. It may be isostructural with the hydrate form along with new patterns containing hydrates and methanol solvate. The slight differences observed could be attributed to variations in residual solvent, lattice changes due to temperature differences, and preferred orientation effects. We have also provided the CIF and CheckCIF files as Supplementary Information for readers’ reference.

The above crystal experiments were directed to check the extensive polymorphism nature of the elacestrant. Elacestrant polymorphism is crucial for determining the stability of the API and guiding the formulation of a robust oral drug product. It ensures consistent dissolution rates, which is critical for achieving the desired bioavailability and therapeutic efficacy.

### 3.3. Extensive Polymorphic Screening Experiment on Crystalline Elacestrant (Form 2/3)

Solubility assessment and polymorph screening were performed using elacestrant with 40 non-conventional solvents, with subsequent treatment of slow cooling, maturation, and/or slow evaporation following solvent-mediated process for crystalline and amorphous forms. Further extensive polymorph screening was performed on only the crystalline form material, polymer templating (12 polymers, 3 solvents), solvent-drop grinding (36 solvents), and anti-solvent addition (12 anti-solvents, 2 solvents).

#### 3.3.1. Solubility Assessment and Polymorph Screening

Polymorph studies were performed using crystalline form material (Form 2/3) as the input material with 40 neat solvent systems to isolate new crystalline polymorphs, hydrates, or solvates of the API. Since polymorphism studies had been performed in the previously published study for elacestrant in common Class 2/Class 3 solvents [24], the solvents selected in this screen were less conventional to increase the chance of identifying additional polymorphs.

The solubility assessment procedure was selected for the 10–50 vol. of solvent at 25 °C and 50 vol. at 50 °C for the crystalline form. Solubility assessments on elacestrant showed low solubility in all organic solvents tested, except benzyl alcohol, ethylene glycol hexafluoropropan-2-ol (HFIP), and trifluoroethanol (TFE), as shown in Table S3. The resulting solids collected from the solubility assessment procedure generated five different XRPD patterns, of which three were new. New pattern A and Form 3 were observed in several solvents, and a range of solid-state techniques characterized a mixture and representative crystalline samples of each. New pattern C and the potential new polymorph plus degradant pattern D were only observed in 1 or 2 solvents, as shown in Table S4 and Figure S4.

#### 3.3.2. Polymer Template Crystallizations

Polymer template crystallization influences crystallization and provides an alternative route to screen for new polymorphs. Depending on the polymer solubility in a selected solvent, it can provide a foreign surface upon which to crystallize or alter solution parameters, e.g., API solubility, viscosity, etc. Suitable solvents for templating experiments were selected based on the known solubility of elacestrant and its relevance to process development (e.g., ethanol, methanol) or if they produced new polymorphs during screening studies (chloroform). All solvents exhibited good compound solubility at suitable volumes and moderate volatility for slow evaporation. A range of up to 12 polymers were utilized to provide templates for crystallization screening.

Polymer template crystallization using the three solvent systems, in the presence of up to 12 different polymers (with varying functional groups and structures), did not produce material exhibiting any additional new XRPD pattern. Form 1 was predominant from templating experiments in the presence of ethanol, and Form 2/3 mixtures, Form 3, or pattern E were produced in the presence of methanol. No crystalline material was isolated from templating experiments in chloroform due to the high solubility of elacestrant in this solvent. The screening procedure and results are shown in Table S5, and supportive XRPD graphs are provided in Figure S5.

#### 3.3.3. Solvent-Drop Grinding Experiments

Solvent-drop grinding experiments were conducted using crystalline material to discover new polymorphs further. The solid was wet with a solvent drop and ground in a Fritsch planetary ball mill.

Solvent-drop grinding experiments using 36 solvent systems, including process-relevant solvents (EtOH, EtOAc, MeOH, iPrOAc), did not produce material exhibiting any additional new XRPD patterns. Most samples were not pure forms, and the XRPD diffractograms were a mixture of XRPD patterns, e.g., Pattern A + Form 2/3. Importantly, the desired form, Form 1, was produced by solvent-drop grinding in ethanol. However, grinding in ethyl acetate or isopropyl acetate produced a mixture of Patterns A + Form 2/3, and grinding in methanol produced Pattern E. Therefore, limiting the volume of ethyl acetate/isopropyl acetate used in the production of Form 1 may be useful to avoid the crystallization of undesired forms. The detailed results are shown in Table S6, and supportive XRPD graphs are provided in Figure S6.

#### 3.3.4. Anti-Solvent Addition Experiments

Anti-solvents were selected from the solubility assessment of elacestrant (Form 2/3), including those that gave low solubility, exhibited new polymorphs, and did not show significant degradation by HPLC. In addition, ethyl acetate and isopropyl acetate anti-solvents were selected based on their common use in process development. Ethanol and methanol were selected as solvents. Ethanol can be used to obtain Form 1, and methanol is known to favor hydrate formation (Form 3) from water activity experiments conducted during the previous study.

Based on prior water activity experiments, it was found that Form 1 could be obtained from slurring in ethanol in up to 5–10% aq./EtOH. To increase the solubility of crystalline elacestrant, dissolution was performed in EtOH/water (96:4, *v*/*v*). This enabled dissolution in 30 vol. of the solvent system at 50 °C. Antisolvent addition experiments in the presence of ethanol/water (96:4) mainly produced the expected Form 1. Two new XRPD patterns were identified during anti-solvent addition in EtOH/water (96:4) when using chloroform (denoted Pattern F) and when using 1,2-dichloroethane or butan-1-ol (denoted Pattern G). In addition, solids were obtained from anisole and chlorobenzene with XRPD patterns similar to Form 3.

The same anti-solvents were added to methanol using 5 vol. of solvent at 50 °C by different ratios. Experiments in the presence of methanol mostly produced material exhibiting a new XRPD pattern, denoted Pattern E.

Pattern A was obtained from MeOH or EtOH/water (96/4) in the presence of 1,2-dimethoxyethane or other anti-solvents when analyzed damp. This material is converted to Form 1 or Pattern E on drying. The polymorphs screening procedure and results are shown in Table S7, and supportive XRPD graphs are provided in Figure S7.

### 3.4. Polymorphic Screening Experiments on Amorphous Elacestrant

Amorphous samples were generated by lyophilization of elacestrant using the procedure in Section 2.5.1. Solubility assessments of amorphous elacestrant showed low solubility in all organic solvents explored, except for benzyl alcohol, ethylene glycol, HFIP, and TFE Table S8. In total, four different XRPD patterns were identified. Pattern A and Form 3 were observed in several solvents, often as a range of solid-state techniques characterized by a mixture and representative crystalline samples. Patterns C and D were only observed in 1 or 2 solvents and may exist as mixtures with other forms. Pattern C was only observed as a mixture with different patterns when starting from amorphous material. HPLC purity analysis of Pattern D assay found ~78% of crystal materials, which showed significant degradation. This was considered a degradant polymorphic form and hence was not considered a new pattern due to its highly degradant form. The polymorphs screening procedure and results are shown in Table S9, and supportive XRPD graphs are in Figure S8.

**Figure 4:**
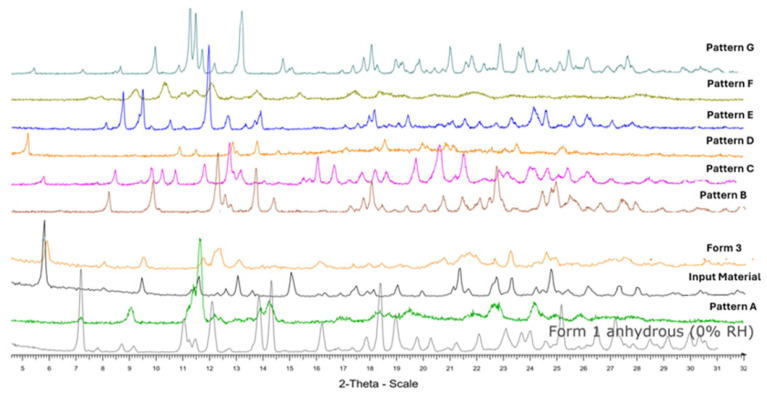
XRPD generated new patterns.

**Figure 5:**
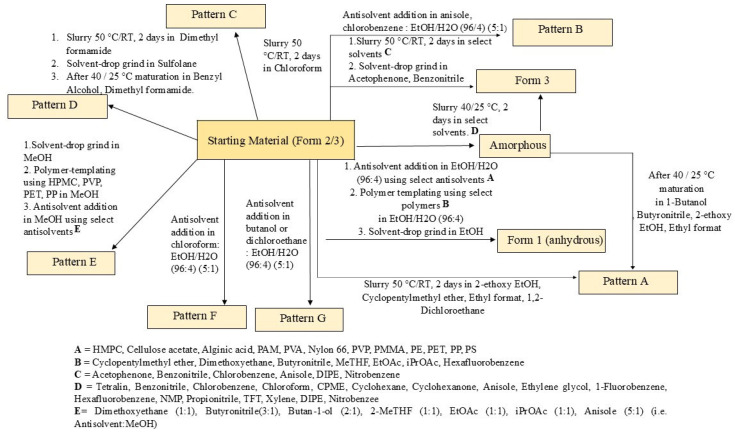
Process diagram of generated polymorphs.

In summary, new patterns A, B, C, E, F, and G were generated throughout the extensive screening process. The most frequently obtained pattern was A, which exhibited thermal behavior similar to Form 1. Pattern C obtained from the chloroform on one occasion was also tentatively assigned as a dihydrate form. Pattern E was found from the antisolvent addition experiment with methanol (dihydrate form); pattern F was obtained by adding chloroform antisolvent to the solution of ethanol/water (96:4), which was assigned as a hemihydrate form (poorly crystalline), and Pattern G produced by adding butan-1-ol or 1,2-dichloroethane to a solution of elacestrant in ethanol/water (96:4), which also assigned as a hemi hydrate form shown as Table 6 and the XRPD of the all-new generated pattern A, B, C, D, E, F, and G, as shown in Figure 4. Lastly, A comprehensive generation process diagram of the elacestrant polymorphsim is shown as Figure 5.

### 3.5. Solid State Characterization of the Generated New Patterns

Representative crystalline samples of Pattern A, B, C, E, F, and G generated from screening using crystalline and amorphous forms of the elacestrant were characterized by solid-state techniques. The solid-state was characterized as the results of XPRD, NMR, DSC, TGA, IC, and HPLC, and 1-week samples of XRPD stability were compared to the characteristics of Form (2/3) and Form 1 (desired form) mentioned in Table 7.

**Figure 6:**
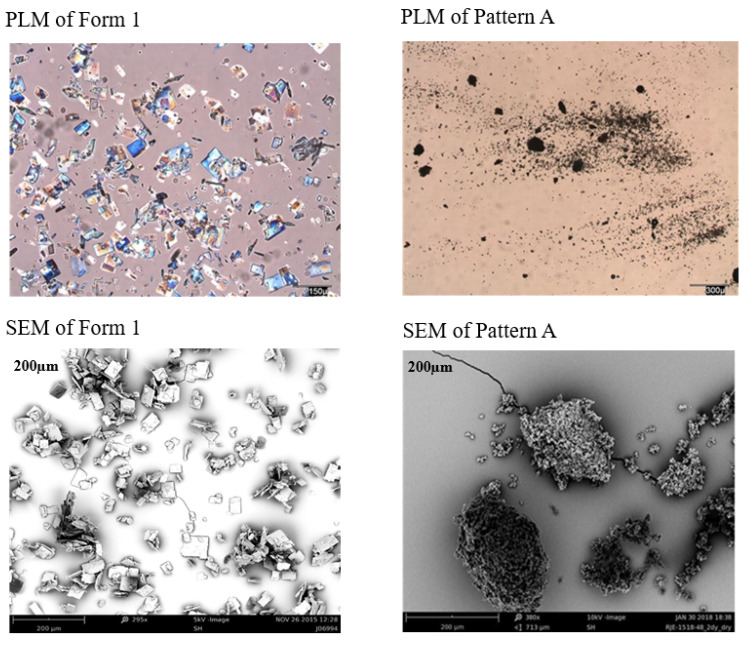
PLM and SEM images of Form 1 and Pattern A.

As per Figure S9I, Pattern A material exhibited the highest melt (225–226 °C, onset) confirmed by DSC. TGA indicated ~0.8% weight loss (RT-90 °C) and 0.7% (90–180 °C) in one sample, while Pattern A, another representative sample, showed 0.5% loss (RT-80 °C) mentioned in Table 7. Pattern A showed the most promising characterization like desired Form 1, but as per Figure 6, PLM and SEM images showed that Pattern A is less crystal than Form 1.

As per Figure S9II, Pattern B was like Form 3 (hydrate) and contained ~5% wt. water (~2 mol eq.) differed subtly by XRPD from the Form 3 shown in Table 7. Pattern C was only observed in chloroform, and TGA indicated 5.2% wt. loss from RT to 100 °C and 1.2% wt. loss from 140 to 190 °C, as shown in Figure S9III. So, Pattern C was expected to not be a chloroform solvate due to no significant loss up to 61 °C (chloroform BP) and compared the TGA-DSC profile with input and starting materials as shown in Figure S1B and found thermal behaviors of the input/starting in our previous research [24], but had a significant water content (5.2% wt, ~2 mol eq.). TGA showed a slight 1.2% weight loss, while DSC indicated peaks at 153.8 °C (onset, endo) and 170.3 °C (peak, exo), reflecting anhydrous form melting/recrystallization. It can be concluded that Pattern A showed the behavior of Form 1, shown in Figure S9IV Pattern B is a hydrate and is similar to Form 3, and Pattern C showed water loss, assigned as a dihydrate form.

As per Figure S9V, Pattern E contained ~5.5% water (~2 mol eq.) and residual methanol. Pattern F was only obtained in chloroform/ethanol–water (96:4) (antisolvent/solvent ratio—5:1) and exhibited similar thermal events to Pattern C but had lower energy melt/recryst. events but showed a total mass loss of 1.6% by TGA (equating to ~0.5 mol eq. water), as shown in Figure S9VI. Pattern G was observed only in the antisolvent addition process by adding antisolvent, butane-1-ol, and butyronitrile, as shown in Table S7, and only showed thermal events relating to solvent loss prior to a melt consistent with Form 1. It can be concluded that Pattern B shows the same thermal behavior as Form 3. Pattern E’s thermal behavior and water content are similar to Pattern C, with melt event onset at ~150 °C, exothermic peak at 170–180 °C, and melt consistently with Form 1. Pattern G thermal, as shown in Figure S9VII and NMR, shows high solvent content (on surface or bound) and melts consistently with Form 1.

Pattern A is similar to Form 1 but exhibits lower crystallinity and converts towards Form 3 at 40 °C/75% RH, as shown in Figure S10. The thermal analysis of Patterns B, C, E, and F showed endothermic–exothermic events (at onset between 136 and 184 °C), indicating a form conversion prior to melting at 205–222 °C (onset). This melt is consistent with Form 1 or Pattern A, suggesting that these materials convert to Form 1 or Pattern A before melting. Pattern G does not show an endothermic–exothermic event, but after desolvation, the material melts at 222 °C (onset). The detailed characterization of the newly generated Patterns of B, E, F, and G was compared with the input materials and Form 1 (desired form) shown in Table 8.

### 3.6. Determination of the Unit Cell Dimensions and the Space Group of the Generated New Patterns

X-ray crystal diffraction determines crystal structures precisely, such as unit cell dimensions, which are provided at three lengths (a, b, c), defining the smallest repeating unit, which provides the idea of the crystal. The diffraction pattern’s symmetry also reveals the defined space group among the existing 230 space groups, which dictates molecular packing within the crystal [45,46]. As a result, to obtain more crystal/lattice information, we determined the unit cell dimension and space group following the Rietveld refine method using the Profex 5.4.1 software [47,48,49]. Rietveld procedure integrated the intensity with the XRPD peaks. Figure S11 (A–J) provided the individual XRPD patterns with intensity scale for the form 1, input material, form 3, and newly generated polymorphs [50,51]. Initially, Profex software identified the phase of the XRPD patterns and found the suitable phase for running the refinement, which provided the unit cell dimension as well as determined the space group of Form 1, starting materials, Form 3, newly generated new patterns of A, B, C, E, F, G (including degradant Pattern D) as shown in Table 9. As shown in Table 9, Form 1, newly generated Pattern D, Pattern F, and Pattern G showed the hexagonal crystal formation. Additionally, Pattern A, Pattern B, Pattern C, and Pattern D showed the trigonal shape of the crystal, with only starting materials, and Form 3 showed the triclinic crystal. This data set provides insightful crystal information about the newly generated polymorphic form of the elacestrant for further study and scale-up.

## 4. Conclusions

A total of six new XRPD patterns/polymorphs were identified from all screening experiments, denoted as Pattern A, B, C, E, F, and G, in addition to the starting material. Pattern D, initially considered a new crystalline material, was later confirmed as a degradant. Pattern A exhibited behavior similar to Form 1 but less crystalline than Form 1, while Pattern B was identified as the hydrate of Form 3 but differed subtly in the XRPD. Pattern C, with water loss, was assigned as a dihydrate form. Thermal analysis of Patterns B, C, E, F, and G indicated an endothermic–exothermic event (136–184 °C) before melting (205–222 °C), consistent with Form 1 or Pattern A. Pattern G, lacking the endothermic–exothermic event, melted at 222 °C after desolvation. Solid-state characterization identified Patterns A, C, and E as crystalline candidates to select from for further in-depth analysis, and unit cell dimensions, space group, and shape provided insightful information about the crystal.

## Figures and Tables

**Table 1:** The molecular properties chart of the elacestrant dihydrochloride.

Properties	Value
Molecular Structure	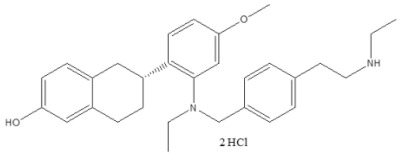
Molecular Weight	531.6 g/mol
pKa	9.8 (BH^+^, aliphatic amine)
cLogP	6.8
LogD	3.6
Hydrogen Bond Donor Count	4
Hydrogen Bond Acceptor Count	4
Rotatable Bond Count	10

**Table 2:** HPLC method to determine chemical purity.

Test Parameter	Result/Value
Method	Reverse phase (gradient elution)
Preparation of samples	0.5–0.7 mg/mL using water:acetonitrile (1:1)
Column type	C18 Supelco Ascentis Express, 100× (4.6 mm, 2.7 μm)
Temperature of Column (°C)	25
Injection volume (µL)	2 or 4
Wavelength (nm)	255
Bandwidth (nm)	90
Rate of Flow (mL per min)	2
Phase A	Water containing 0.1% TFA
Phase B	Acetonitrile containing 0.085% TFA

**Table 3:** Details of the IC methods.

Test Parameter	Result/Value
Method type	Anion exchange
Type of Column	Metrosep A Supp 5–150 (4.0 × 150 mm)
Temperature of Column (°C)	Room temperature
Injection volume (µL)	Various
Type of Detection	Conductivity detector
Rate of flow (mL/min)	0.7
Eluent	Sodium Carbonate (3.2 mM), Sodium Hydrogen Carbonate (1.0 Mm) in an aqueous solution of 5% acetone.

**Table 4:** Details of the hydrogen bonding interactions.

D-H···A	d(D-H) Å	d(H···A) Å	d(D···A) Å	<(DHA)°
O1-H1···Cl2 ^#1^	0.78 (4)	2.34 (4)	3.110 (3)	168 (4)
N1-H1A···Cl2 ^#2^	0.89 (4)	2.43 (4)	3.246 (3)	153 (3)
N2-H2B···Cl1	0.95 (5)	2.17 (5)	3.106 (3)	170 (4)
N2-H2A···Cl2	0.89 (5)	2.26 (5)	3.121 (3)	163 (4)
O3-H3A···Cl1	0.88 (6)	2.34 (6)	3.174 (4)	158 (5)

^#1^ x−1,y,z ^#2^ −x + 1,y + 1/2,−z + 1/2.

**Table 5:** Sample details and crystal data for elacestrant methanol solvate.

Sample Details	Crystal Data
Compound	Elacestrant methanol solvate
Crystallization solvents	Methanol
Crystallization method	slow cooling
Empirical formula	C32.5H44Cl2N2O4.50
Formula weight	605.59
Temperature	100(2) K
Wavelength	1.54184 Å
Crystal size	0.380 × 0.380 × 0.050 mm
Crystal habit	colorless plate
Crystal system	Orthorhombic
Space group	*P*2_1_2_1_2_1_
Unit cell dimensions	*a* = 8.93126(11) Å α = 90°
	*b* = 11.70665(14) Å β = 90°
	*c* = 31.4469(4) Å γ = 90°
Volume	3287.93(7) Å3
Z	4
Density (calculated)	1.223 mg/m^3^
Absorption coefficient	2.086 mm^−1^

**Table 6:** Summary of all generated new patterns.

Pattern	Description	Conclusion
Starting /Input Material	Elacestrant supplied as a mixture of Form 2 and Form 3	Mixture of Form 2 and Form 3
A	Obtained frequently, had the highest melt temperature at 225–226 °C (onset)	New Polymorph
B	Similar to Form 3 but with a unique XRPD pattern	New Polymorph
C	Obtained from chloroform on one occasion and is tentatively assigned as a dihydrate form	New Polymorph
D	Obtained on two occasions using dimethyl formamide and benzyl alcohol, but found to have degraded (78% purity by HPLC)	Potential New Polymorph with Degradation
E	Obtained on numerous occasions from anti-solvent addition experiments with methanol, tentatively assigned as a dihydrate form	New Polymorph
F	Obtained on one occasion by adding chloroform anti-solvent to a solution of elacestrant in ethanol (4% aq.). Tentatively assigned as a hemihydrate form.	New Polymorph
G	Obtained on two occasions by the addition of butan-1-ol or 1,2-dichloroethane to a solution of elacestrant in ethanol (4% aq.). Tentatively assigned as a hemihydrate form, exhibiting a small mass loss prior to a melt consistent with Form 1.	New Polymorph

**Table 7:** Solid-state characterization data for representative crystalline samples of A, C compared to Form 1.

Technique	Elacestrant (Form 2/3 Mixture-Input Material)	Elacestrant (Form 1)	1,2-Dichloroethane (Table S9)	1,2-Dichloroethane (Table S4)	Chloroform (Dry) (Table S4)
XRPD	Input material (Form 2/3 mixture)	Form 1	Crystalline, Pattern A		Crystalline, Pattern C
^1^H-NMR	Consistent with NMR of previous batch Form 2/3 reference material but with 0.08 eq. residual MeOH.	Consistent with structure	Consistent with as supplied. No residual solvent.	Consistent with as supplied, but with 0.1 eq dichloroethane	Consistent with as supplied. No residual solvent.
DSC	Broad endo. RT to 150 °C, minimum at 86.4 °C (peak). Endo. at 163.1 °C (onset), 51 J/g. Sample degrades above 200 °C.	Endotherm at 218 °C(onset) 150.0 J/g(melt)	Endo. At 56.7 °C (peak), 10 J/g. Melt endo. at 225.5 °C (onset), 119 J/g.	Endo. At 50.4 °C (onset), 16 J/g. Melt endo. at 224.9 °C (onset), 116 J/g.	Broad endo at 69.5 °C (onset), 151 J/g. Melt recryst. at 153.8 °C (onset, endo), 20 J/g, 170.3 °C (peak, exo). Melt endo at 219.5 °C (onset), 111 J/g.
TGA	8.4% wt. loss from RT to 150 °C and 0.6% wt. loss from 150 to 190 °C	0.4% weight loss between ambient and 100 °C	0.5% wt. loss from RT to 80 °C	0.8% wt. loss from RT to 90 °C and 0.7% wt. loss from 90 to 180 °C	5.2% wt. loss from RT to 100 °C and 1.2% wt. loss from 140 to 190 °C
IC (adjusted for water/solvent)	1.82 eq.	2.0 eq.	1.84 eq.	1.79 eq.	1.82 eq.
HPLC (%, AUC)	99.2%	99.2%	98.8%	98.2%	97.6%
XRPD post storage at 40 °C/75% RH for 1 week	N/P	Form 1	Pattern A + Form 3	Pattern A + Form 3	Pattern C with increased crystallinity
Comments	Form 2/3 anhydrous/hydrate mixture	Form 1 anhydrous	Similar to Form 1 but exhibits lower crystallinity and converts towards Form 3 at 40 °C/75% RH, as shown in Figure S10		Thermal data shows water loss, melt/recrys. of the anhydrous form and then melt consistent with Form 1

**Table 8:** Solid-state characterization data for representative crystalline samples of B, E, F, and G compared to Form 1.

Technique	Elacestrant (Form 2/3 Mixture-Input Material)	Elacestrant (Form 1)	Anisole _5C_Dry_Table S7	Butyronitrile_5C_Dry_Table S7	EtOAc_5C_Dry_Table S7	Chloroform_5C_Dry_Table S7	Butan-1-ol_5C_Dry_Table S7
XRPD	Input material (Form 2/3 mixture)	Form 1	Crystalline, Pattern B	Pattern E		Poorly crystalline, Pattern F	Crystalline, Pattern G
^1^H-NMR	Consistent with NMR of previous reference batch, but with 0.08 eq. residual MeOH.	Consistent with structure	Consistent with as supplied, no residual solvent observed	Consistent with as supplied, but with 0.04 eq. residual MeOH	Consistent with as supplied, but with 0.06 eq. residual MeOH and trace EtOAc	Consistent with as supplied, no residual solvent observed	Consistent with as supplied, but with 0.77–0.83 eq. butan-1-ol, 0.18–0.28 eq. EtOH 0.06 eq. MeOH
DSC	Broad endo. RT to 150 °C, minimum at 86.4 °C (peak). Endo. at 163.1 °C (onset), 51 J/g. Sample degrades above 200 °C.	Endotherm at 218 °C (onset) 150.0 J/g (melt)	Broad endo at 37.9 °C (onset), 73 J/g. Meltrecryst. at 183.4 °C (onset, endo), 52 J/g, 199.9 °C (peak, exo). Melt endo at 209.4 °C (onset), 48 J/g.	Broad endo from RT to 170 °C, with endo/exo event at 150.1 °C (onset, endo), 116 J/g, °C (peak, 184.0 exo) and melt endo at 205.0 °C (onset), 39 J/g	Broad endo from RT to 170 °C, with endo/exo event at 154.2 °C (onset, endo), 106 J/g, 185.6 °C (peak, exo) and melt endo at 205.4 °C (onset), 19 J/g	Broad endo at 35.2 °C (onset), 13 J/g. small endo/exo event at 135.5 °C (onset, endo), 4 J/g, 158.5 °C (peak, exo) and melt endo at 222.0 °C (onset), 123 J/g	Sharp endo at 99.6 °C (onset), 73 J/g, and melt endo at 222.3 °C (onset), 90 J/g
TGA	8.4% wt. loss from RT to 150 °C and 0.6% wt. loss from 150 to 190 °C	0.4% wt. loss from RT to 100 °C	3.1% wt. loss from RT to 90 °C and 1.8% wt. loss from 170 to 210 °C	5.5% wt loss from RT to 170 °C	5.3% wt loss from RT to 170 °C	1.3% wt loss from RT to 100 °C and 0.3% wt loss from 110 to 180 °C	1.9% wt loss from RT to 90 °C and 8.1% wt loss from 90 to 150 °C
HPLC (%, AUC)	99.2%	99.2%	98.9%	99.3%	99.4%	99.1%	98.8%
XRPD post storage at 40 °C/75% RH 1 week.	Not performed	Form 1	Pattern A	Pattern E	Pattern E	Form 1	Form 3
Comments	Form 2/3 anhydrous/hydrate mixture	Form 1 anhydrous	Thermal consistent with Form 3, hydrated form	Thermal behavior and water content very similar to Pattern C, with melt/recryst. event onset at ~150 °C, exo peak at 170–180 °C, and melt consistent with Form 1		Similar to Pattern C but weaker thermal events (both show melt/recryst. prior to melt consistent with Form 1)	Thermal and NMR show high solvent content (on surface or bound) and melt consistent with Form 1

**Table 9:** Determination of the unit cell dimensions and the space group of Form 1, starting/input material, Form 3, and generated new patterns.

Software: Profex 5.4.1										
Method: Rietveld Refinement										
Parameters	Form 1	Starting /Input Material	Form 3	Pattern A	Pattern B	Pattern C	Pattern D	Pattern E	Pattern F	Pattern G
Phase	BGMN (SiC6H)	BGMN (Kaolinite)	BGMN (Kaolinite)	BGMN (SiC15R)	BGMN (Magnesite)	BGMN (Magnesite)	BGMN (SiC6H)	BGMN (Hematite)	BGMN (SiC6H)	BGMN (SiC6H)
Unit cell dimensions	*a* = 3.081 Å	*a* = 5.16 Å	*a* = 5.16 Å	*a* = 3.07 Å	*a* = 4.63 Å	*a* = 4.63 Å	*a* = 3.08 Å	*a* = 5.04 Å	*a* = 3.08 Å	*a* = 3.08 Å
	*b* = undf	*b* = 5.16 Å	*b* = 8.95 Å	*b* = undf	*b* = undf	*b* = undf	*b* = undf	*b* = undf	*b* = undf	*b* = undf
	*c* = 15.17 Å	*c* = 7.40 Å	*c* = 7.40 Å	*c* = 37.70 Å	*c* = 15.01 Å	*c* = 15.01 Å	*c* = 15.12 Å	*c* = 13.77 Å	*c* = 15.12 Å	*c* = 15.12 Å
	α = β = 90°, γ = 120°	α = 91°, β = 105°, γ = 89°	α = 91°, β = 105°, γ = 89°	α = β = 90°, γ = 120°	α = β = 90°, γ = 120°	α = β = 90°, γ = 120°	α = β = 90°, γ = 120°	α = β = 90°, γ = 120°	α = β = 90°, γ = 120°	α = β = 90°, γ = 120°
Space group	186 Hermann Mauguin = P6_3mc	1 Hermann Mauguin = P1	1 HermannMauguin = P1	160 HermannMauguin = R3m	167 HermannMauguin = R-32/c	167 HermannMauguin = R-32/c	186 HermannMauguin = P6_3mc	167 HermannMauguin = R-32/c	186 HermannMauguin = P6_3mc	186 HermannMauguin = P6_3mc
Crystal Shape	Hexagonal	Triclinic	Triclinic	Trigonal	Trigonal	Trigonal	Hexagonal	Trigonal	Hexagonal	Hexagonal

undf = undefined.

## Data Availability

The data will be made available on request.

## Supplementary Materials

The following supporting information can be downloaded at: https://www.mdpi.com/article/10.3390/pharmaceutics17060745/s1, Figure S1: (I) NMR of starting material (II). TGA and DSC of starting material. Figure S2: XRPD diffractogram elacestrant methanol solvate for comparison with experimental XRPD diffractograms of elacestrant Form 1, Form 2, and Form 3. Figure S3: XRPD diffractogram elacestrant methanol solvate (black) for comparison with experimental XRPD diffractograms of elacestrant Form 3, Pattern C, and Pattern E. Figure S4: (I–VII) XRPD diffractograms of solids obtained after 50 °C/RT maturation or cooling of crystalline material in different solvents. XRPD diffractograms are shown for ‘damp’ and then ‘dry’ samples. Figure S5: (I) XRPD of solids obtained after polymer templating experiments (EtOH) (4% aq.); (II–III) XRPD of solids obtained after polymer templating experiments (MeOH); (IV) XRPD of solids obtained after polymer templating experiments (chloroform). Figure S6: (I–IV) XRPD of solids obtained after solvent-drop grinding experiments. Figure S7: (I) XRPD of solids obtained at 50 °C upon anti-solvent; (II–VII) XRPD solids obtained at 5 °C upon anti-solvent are shown damp, then dry. Figure S8: (I–VIII) XRPD diffractograms of solids obtained after cooling or maturation of amorphous material in different solvents. XRPD diffractograms are shown for ‘damp’ and then ‘dry’ samples. Figure S9: DSC and TGA of the (I) Pattern A, (II) Form B, (III) Form C, (IV) Form 1, (V) Form E, (VI) Form F, (VII) Form G. Figure S10: XRPD diffractogram of Pattern A pre- and post-storage at 40 °C/75% RH for 1 week. Figure S11: Individual XRPD with intensity peak of (A) Form 1; (B) starting/input materials; (C) Form 3 (D–J), Pattern A-G. Table S1: Experimental conditions and observations from single-crystal growth experiments. Table S2: Data collection and structure refinement for elacestrant methanol solvate. Table S3: Solubility assessment by using different solvents of crystalline form. Table S4: Polymorph screening experiments on samples of crystalline material post-solubility assessment. Table S5: Polymer template crystallization using crystalline material. Table S6: Solvent-drop grinding experiments using crystalline material. Table S7: Anti-solvent addition experiments using crystalline material. Table S8: Solubility assessment is performed using different solvents of amorphous form. Table S9: Polymorph screening experiments on samples of amorphous material post-solubility assessment.
