# Supplementary material for: Novel Polymorphic Patterns for Elacestrant Dihydrochloride
Source: Pharmaceutics. 2025 Jun 5;17(6):745. doi: 10.3390/pharmaceutics17060745 (PMC12197126; doi:10.3390/pharmaceutics17060745)

SUPPLEMENTARY INFORMATION

Novel Polymorphic Patterns for Elacestrant Dihydrochloride

Zia Uddin Masum,[a] P. Grant Spoors,[b] Matt D. Burke,[b*] Vivek Gupta[a*]

[a] Department of Pharmaceutical Sciences
College of Pharmacy and Health Sciences
St. John's University
8000 Utopia Parkway, Queens, NY 11439, USA

[b] Stemline Therapeutics, Inc., A Menarini Group Company
750 Lexington Avenue, 4th Floor
New York, NY 10022, USA

S2.2: Instruments & Methods

S2.2.1 X-Ray Powder Diffraction (XRPD):

S2.2.1.1 Bruker AXS D8 Advance

X-Ray Powder Diffraction patterns were also collected on a Bruker D8 diffractometer (Bruker, Madison, WI, USA) using Cu Kα radiation (40 kV, 40 mA), θ - 2θ goniometer, and divergence of V4 and receiving slits, a Ge monochromator and a Lynxeye detector. The data were analyzed and presented using Diffrac *Plus* XRD Commander & Diffrac *Plus* EVA v15.0.0.0 software.

Samples were run under ambient conditions as flat plate specimens using powder as received. The sample was prepared on a polished, zero-background (510) silicon wafer by gently pressing onto the flat surface or packed into a cut cavity. The sample was rotated in its plane.

S2.2.1.2 PANalytical Empyrean

X-Ray Powder Diffraction patterns were collected on a PANalytical Empyrean diffractometer using Cu Kα radiation (45 kV, 40 mA) in transmission geometry. A 0.5° slit, 4 mm mask, and 0.04 rad Soller slits with a focusing mirror were used on the incident beam. A PIXcel3D detector, placed on the diffracted beam, was fitted with a receiving slit and 0.04 rad Soller slits. The software used for data collection was X'Pert Data Collector using X'Pert Operator Interface. The data were analyzed and presented using Diffrac *Plus* EVA software.

Samples were prepared and analyzed in either a metal or Millipore 96 well-plate in transmission mode. A transparent X-ray film was used between the metal sheets on the metal well plate, and powders (approximately 1–2 mg) were used as received. The Millipore plate was used to isolate and analyze solids from suspensions by adding a small amount of rest directly to the plate before filtration under a light vacuum. The scan mode for the metal plate used the gonio scan axis, whereas a 2θ scan was utilized for the Millipore plate.

S2.2.2 TA Instruments Discovery DSC:

DSC data were collected on a TA Instruments Discovery DSC equipped with a 50-position auto-sampler. Typically, 0.5-3 mg of each sample was placed in a pin-holed aluminum pan and heated at 10 °C/min from 25 °C to 300 °C. A purge of dry nitrogen at 50 ml/min was maintained over the sample. The data were analyzed using TRIOS or Universal Analysis software.

S2.2.3 TA Instruments Discovery TGA

TGA data were collected on a TA Instruments Discovery TGA equipped with a 25-position auto-sampler. Typically, 5 - 10 mg of each sample was loaded onto a pre-tared aluminum DSC pan and heated at 10 °C/min from ambient temperature to 350 °C. A nitrogen purge at 25 ml/min was maintained over the sample. The data were analyzed using TRIOS or Universal Analysis software.

Table S1 : Experimental conditions and observations from single crystal growth experiments.

Solvent	Quantity								after treatmentObservation	Further treatment	Observation after treatment	Further treatment	Observation after treatment
	10 vol	10 vol	20 vol	30 vol	40 vol	60 vol	80 vol	100 vol					
Ethylene glycol	X	X	X	P	-	-	-	-	Clear blue solution	Cooled	Clear blue solution	Warmed to room temperature and allowed to evaporate	NFA
Methanol	P	P	-	-	-	-	-	-	Clear solution	Cooled	**Cubic crystals**	Warmed to room temperature	**Cubic crystals Submitted to SCXD**
Ethanol	X	X	X	X	X	X	X	X	Thin suspension	Maturation	Thin suspension	Liquors split and evaporated, cooled and kept	NFA
Water:Ethanol$_{(1:99)}$	X	X	X	X	X	X	X	P	Clear solution	Cooled	Plate shaped crystals	Warmed to room temperature	Plate crystals
Water:Ethanol$_{(2:98)}$	X	X	X	X	X	X	P	-	Clear solution	Cooled	Plate shaped crystals	Warmed to room temperature	Plate crystals
Water:Ethanol$_{(3:97)}$	X	X	X	X	X	P	-	-	Clear solution	Cooled	Small cubic crystals	Warmed to room temperature	Cubic crystals
Water:Ethanol$_{(1:9)}$	P	-	-	-	-	-	-	-	Clear solution	Cooled	Yellow solution	Warmed to room temperature and allowed to evaporate	Clear solution
Benzyl alcohol	P	P	-	-	-	-	-	-	Clear solution	Cooled	Clear solution	Warmed to room temperature and allowed to evaporate	Clear solution
Hexafluorpropanol	P	-	-	-	-	-	-	-	Black solution	Cooled	Black solution	Warmed to room temperature and allowed to evaporate	Clear solution

Trifluoroethanol	P	-	-	-	-	-	-	-	Clear solution	Cooled	Cloudy white suspension	Warmed to room temperature and allowed to evaporate	Clear solution
Water	P	P	-	-	-	-	-	-	Clear solution	Cooled	**Crystalline needles and laths**	Warmed to room temperature	**Needle/rod crystals Submitted to SCXD**
DMSO	P	-	-	-	-	-	-	-	Clear solution	Cooled	Frozen	Warmed to room temperature and allowed to evaporate	Clear solution
1,2-dichloroethane	X	X	X	X	X	X	X	X	Thin suspension	Maturation	Thin suspension	Liquors split and evaporated, cooled and kept	NFA
Nitrobenzene	X	X	X	X	X	X	P	P	Thins suspension	Maturation	Thins suspension	Liquors split and evaporated, cooled and kept	NFA

Key: P = solution, - = not performed, X = suspension

Table S2: Data collection and structure refinement for Elacestrant methanol solvate.

Diffractometer	SuperNova, Dual, Cu at zero, Atlas
Radiation source	SuperNova (Cu) X-ray Source, CuKα
Data collection method	omega scans
Theta range for data collection	4.029 to 70.174°
Index ranges	$-10 \le h \le 10$, $-13 \le k \le 14$, $-38 \le l \le 38$
Reflections collected	59685
Independent reflections	6211 [R(int) = 0.0483]
Coverage of independent reflections	99.9 %
Variation in check reflections	n/a
Absorption correction	Semi-empirical from equivalents
Max. and min. transmission	1.00000 and 0.60532
Structure solution technique	Direct method
Structure solution program	SHELXTL
Refinement technique	Full-matrix least-squares on *F*2
Refinement program	SHELXL-2013
Function minimized	$\sum w(Fo^2\text{-}Fc^2)2$
Data/restraints/parameters	6211 / 51 / 413
Goodness-of-fit on F^2	1.074
▲/σ_{max}	0.000
Final R indices Data; $I>2\sigma$ (I)	R1 = 0.0412, wR2 = 0.1116
	R1 = 0.0424, wR2 = 0.1129
Weighting scheme	$w=1/[\sigma^2(F_o^2)+(0.0599P)^2+2.3559P]$
	where $P=(F_o^2+2F_c^2)/3$
Absolute structure parameter	-0.018(5)
Extinction coefficient	n/a
Largest diff. peak and hole	0.691 and -0.415 *e*Å-3

Table S3: Solubility assessment by using different solvents of Crystalline form

Solvent	Vol of solvent					
	10	20	30	40	50	50
	25°C					50°C
Hexane	X	X	X	X	X	X
Tetralin	X	X	X/+/-	+/-	+/-	+/-
Acetic acid	X	X	X	X	X	X/+/-
Acetophenone	X	X	X/+/-	+/-	+/-	+/-
Benzonitrile	X	X/+/-	X/+/-	X/+/-	+/-	+/-
Benzyl alcohol	P					
1-Butanol	X	X	X	X	X	X
Butyronitrile	X	X	X	X	X	X
Chlorobenzene	X	X	X/+/-	+/-	+/-	+/-
Chlorobenzotrifluoride	X	X	X	X	X/+/-	X/+/-
Chloroform	X	X	X	X	X	X/+/-
Cyclopentylmethyl ether	X	X	X	X	X	X
Cyclohexane	X	X	X	X	X	X
Cyclohexanone	X	X	X	X	X	X
1,2-Dichlorobenzene	X	X/+/-	X/+/-	+/-	+/-	+/-
1,2-Dichloroethane	X	X	X	X	X	X
Dimethoxyethane	X	X	X	X	X	X
Dimethylacetamide	X	X	X	X/+/-	+/-	P
Anisole	X	X	X/+/-	+/-	+/-	+/-
Ethylene glycol	P					
1-Fluorobenzene	X	X	X	X/+/-	X/+/-	X/+/-
Glycerol	X	X/+/-	X/+/-	+/-	+/-	P
Hexafluorobenzene	X	X	X	X	X	X
Hexafluoropropan-2-ol	P					
Methyl-THF	X	X	X	X	X	X
NMP	X	X	X	X/+/-	+/-	P
Perfluorohexane	X	X	X	X	X	X

Propionitrile	X	X	X	X	X	X
Sulfolane	X	X	X	X	X/+/-	X/+/-
Trifluoroethanol	P					
Trifluorotoluene	X	X	X	X	X	X/+/-
o-Xylene	X	X	X	X/+/-	+/-	+/-
Cumene	X	X	X	+/-	+/-	+/-
Cyclohexanol	X	X	X	X	X	+/-
Dimethyl formamide	X	X	X	X	X	+/-
Dimethyl carbonate	X	X	X	X	X	X
Ethyl formate	X	X	X	X	X	X
Diisopropyl ether	X	X	X	X	X	X
Nitrobenzene	X	X	+/-	+/-	+/-	+/-
2-ethoxyethanol	X	X	X	X	X	X

Key: N/P = not performed, X = suspension, +/- = thin suspension, P = solution

Table S4: Polymorph screening experiments on samples of crystalline material post-solubility assessment

Solvent	After 50 °C / RT maturation	After 5 °C cool	Evaporation	XRPD 'damp'	XRPD 'dry'
Hexane	Suspension	N/P	N/P	Consistent with input material	Consistent with input material
Tetralin	Thin suspension	N/P	N/P	Consistent with input material (poorly cryst.)	Consistent with input material
Acetic acid	Suspension	N/P	N/P	Consistent with input material	Consistent with input material
Acetophenone	Suspension	N/P	N/P	Poorly cryst.	Form 3
Benzonitrile	Suspension	N/P	N/P	Form 3	Form 3
Benzyl alcohol	N/P	Solution	Yellow Solution	N/P	N/P
1-Butanol	Suspension	N/P	N/P	Pattern A	Pattern A + Form 3
Butyronitrile	Suspension	N/P	N/P	Pattern A + Form 3	Pattern A + Form 3
Chlorobenzene	Thin Suspension	N/P	N/P	Pattern A + Form 3	Form 3
Chlorobenzotrifluoride	Suspension	N/P	N/P	Consistent with input material	Consistent with input material
Chloroform	Suspension	N/P	N/P	Poorly cryst.	Pattern C
Cyclopentylmethyl ether	Suspension	N/P	N/P	Pattern A	Pattern A + 2 peaks
Cyclohexane	Suspension	N/P	N/P	Consistent with input material	Consistent with input material
Cyclohexanone	Thin Suspension	N/P	N/P	Pattern A	Pattern A + Form 3 + 2 peaks
1,2-Dichlorobenzene	Thin Suspension	N/P	N/P	No peaks	Consistent with input material
1,2-Dichloroethane	Thin Suspension	N/P	N/P	Pattern A	Pattern A
Dimethoxyethane	Suspension	N/P	N/P	Pattern A + Form 3	Pattern A + Form 3
Dimethylacetamide	N/P	Solution	Solution	N/P	N/P
Anisole	Thin Suspension	N/P	N/P	Pattern A (poorly cryst.)	Form 3
Ethylene glycol	N/P	Solution	Crystals	N/P	N/P
1-Fluorobenzene	Suspension	N/P	N/P	Consistent with input material	Consistent with input material + some Pattern A
Glycerol	N/P	Solution	Ongoing	N/P	N/P
Hexafluorobenzene	Suspension	N/P	N/P	Consistent with input material	Consistent with input material
Hexafluoropropan-2-ol	N/P	Solution	Brown solution	N/P	N/P

Methyl-THF	Suspension	N/P	N/P	Pattern A + Form 3	Pattern A + Form 3
NMP	N/P	Solution	Solution	N/P	N/P
Perfluorohexane	Suspension	N/P	N/P	Consistent with input material	Consistent with input material
Propionitrile	Suspension	N/P	N/P	Pattern A + Form 3	Pattern A + Form 3
Sulfolane	Thin Suspension	N/P	N/P	No peaks	Poorly cryst.
Trifluoroethanol	N/P	Solution	Brown solution	N/P	N/P
Trifluorotoluene	Suspension	N/P	N/P	Consistent with input material	Consistent with input material
o-Xylene	Thin Suspension	N/P	N/P	No peaks	No peaks
Cumene	Thin Suspension	N/P	N/P	Consistent with input material	Consistent with input material
Cyclohexanol	Thin Suspension	N/P	N/P	Amorphous	Amorphous
Dimethyl formamide	Thin Suspension	N/P	N/P	Pattern D	Pattern D
Ethyl formate	Suspension	N/P	N/P	Pattern A	Pattern A
Diisopropyl ether	Suspension	N/P	N/P	Form 3	Form 3
Nitrobenzene	Thin Suspension	N/P	N/P	Form 3	Form 3
2-ethoxyethanol	Suspension	N/P	N/P	Pattern A	Pattern A

Key: N/P = not performed. Data provided in Data Section 3- Polymorph Screening Post Solubility Assessment on Amorphous Material

Table S5: Polymer template crystallization using crystalline material

Solvent	Polymer	M.P. (°C)	Solubility in solvent	After evaporation	XRPD
EtOH/water (96:4)	Hydroxypropylmethyl cellulose (HPMC)	N/A	Soluble	Solid residue	Form 1*
	Cellulose acetate	N/A	Insoluble	Solid residue	Form 1
	Alginic acid sodium salt	N/A	Insoluble	Solid residue	Form 1
	Polyacrylamide (PAM)	N/A	Insoluble	Solid residue	Form 1
	Poly(vinylalcohol) (PVA)	200	Insoluble	Solid residue	Form 1*
	Nylon 66	264	Insoluble	Solid residue	Form 1*
	Poly(vinylpyrrolidone) (PVP)	150-180	Soluble	Solid residue	Form 1*
	Poly(methylmethacrylate) (PMMA)	160	Insoluble	Solid residue	Form 1*
	Polyethylene (PE)	115-135	Insoluble	Solid residue	Form 1
	Poly(ethylene terephthalate) (PET)	250	Insoluble	Solid residue	Form 1
	Polypropylene (PP)	130-170	Insoluble	Solid residue	Form 1*
	Polystyrene (PS)	240	Insoluble	Solid residue	Form 1
MeOH	Hydroxypropylmethyl cellulose (HPMC)	N/A	Soluble	Solid residue	Pattern E
	Cellulose acetate	N/A	Insoluble	Solid residue	Consistent with input material + Form 3
	Alginic acid sodium salt	N/A	Insoluble	Solid residue	Consistent with input material + Form 3
	Polyacrylamide (PAM)	N/A	Insoluble	Solid residue	Consistent with input material + Form 3
	Poly(vinylalcohol) (PVA)	200	Insoluble	Solid residue	Consistent with input material + Form 3
	Nylon 66	264	Insoluble	Solid residue	Consistent with input material + Form 3
	Poly(vinylpyrrolidone) (PVP)	150-180	Soluble	Solid residue	Pattern E

	Poly(methylmethacrylate) (PMMA)	160	Insoluble	Solid residue	Consistent with input material + Form 3
	Polyethylene (PE)	115-135	Insoluble	Solid residue	Consistent with input material + Form 3
	Poly(ethylene terephthalate) (PET)	250	Slight	Solid residue	Pattern E
	Polypropylene (PP)	130-170	Slight	Solid residue	Pattern E
	Polystyrene (PS)	240	Insoluble	Solid residue	Consistent with input material + Form 3
Chloroform	Poly(vinylalcohol) (PVA)	N/A	Insoluble	Solid residue	Amorphous/ polymer peak
	Poly(vinylpyrrolidone) (PVP)	N/A	Soluble	Clear gum	N/P
	Poly(methylmethacrylate) (PMMA)	N/A	Soluble	Clear gum	N/P
	Poly(ethylene terephthalate) (PET)	N/A	Slight	No solids	N/P
	Polystyrene (PS)	200	Soluble	Solid residue	No peaks

*additional peaks, which may be due to residual polymer

Table S6: Solvent-drop grinding experiments using crystalline material

Solvent	After solvent drop grinding	XRPD
Hexane	Solid residue	Consistent with input material
Tetralin	Solid residue	Consistent with input material
Acetic acid	Solid residue	Consistent with input material
Acetophenone	Solid residue	Form 3
Benzonitrile	Solid residue	Form 3
Benzyl alcohol	Solid residue	Pattern D + Form 3
1-Butanol	Solid residue	Pattern A + Form 3
Butyronitrile	Solid residue	Pattern A + Form 3
Chlorobenzene	Solid residue	Consistent with input material
Chlorobenzotrifluoride	Solid residue	Consistent with input material
Chloroform	Solid residue	Consistent with input material
Cyclopentylmethyl ether	Solid residue	Consistent with input material
Cyclohexane	Solid residue	Consistent with input material
Cyclohexanone	Solid residue	Consistent with input material
1,2-Dichlorobenzene	Solid residue	Consistent with input material
1,2-Dichloroethane	Solid residue	Pattern A + Form 3
Dimethoxyethane	Solid residue	Pattern A + Form 3
Dimethylacetamide	Sticky gum	N/P
Anisole	Solid residue	Consistent with input material + Pattern A + Form 3
Ethylene glycol	Solid residue	Consistent with input material
1-Fluorobenzene	Solid residue	Consistent with input material + Pattern A + Form 3
Glycerol	Sticky gum	N/P
Hexafluorobenzene	Solid residue	Consistent with input material
Hexafluoropropan-2-ol	Brown gum	N/P
Methyl-THF	Solid residue	Consistent with input material + Pattern A + Form 3
NMP	Stick gum	N/P

Perfluorohexane	Stick gum	N/P
Propionitrile	Solid residue	Consistent with input material + Form 1
Sulfolane	Solid residue	Pattern D
Trifluoroethanol	Gum	N/P
Trifluorotoluene	Solid residue	Consistent with input material + Pattern A + Form 3
o-Xylene	Solid residue	Consistent with input material + Pattern A + Form 3
EtOH	Solid residue	Form 1
MeOH	Solid residue	Pattern E
EtOAc	Solid residue	Consistent with input material + Pattern A + Form 3
iPrOAc	Solid residue	Consistent with input material + Pattern A + Form 3

Table S7: Anti-solvent addition experiments using crystalline material

Solvent	Vol of solvent to dissolve API at 50 °C	Anti-solvent added	Ratio of anti-solvent: solvent	Observations upon addition at 50°C	After cooling mixture to 5°C	XRPD (50 °C samples)	XRPD 'damp' (5 °C samples)	XRPD 'dry' (5 °C samples)
EtOH/water (96:4)	20	Chloroform	5:1	Thin suspension	Thin suspension	N/P	Pattern F	Pattern F
	20	Cyclopentylmethyl ether	1:1	Suspension	Suspension	Pattern A	Form 1	Form 1
	20	Dimethoxyethane	1:1	Suspension	Suspension	Pattern A	Form 1	Form 1
	30	1,2-Dichloroethane	5:1	Thin suspension	Thin suspension	N/P	Pattern G	Pattern G
	30	Butan-1-ol	5:1	Solution	Thin suspension	N/P	Pattern G	Pattern G
	30	Butyronitrile	1:1	Thin suspension	Thin suspension	N/P	Form 1	Form 1
	30	MeTHF	1:1	Thin suspension	Thin suspension	N/P	Form 1	Form 1
	30	EtOAc	1:1	Thin suspension	Thin suspension	N/P	Form 1	Form 1
	30	iPrOAc	1:1	Thin suspension	Thin suspension	N/P	Form 1	Form 1
	30	Anisole	5:1	Thin suspension	Thin suspension	N/P	Pattern B	Pattern B
	30	Chlorobenzene	5:1	Thin suspension	Thin suspension	N/P	Pattern B	Pattern B
	30	Hexafluorobenzene	2:1	Thin suspension	Thin suspension	N/P	Form 1	Form 1
MeOH	5	Chloroform	5:1	Solution	Thin suspension	N/P	No peaks	Poorly cryst.
	5	Cyclopentylmethyl ether	1:1	Suspension	Suspension	Consistent with input material	Consistent with input material	Consistent with input material
	5	Dimethoxyethane	1:1	Suspension	Suspension	Pattern A	Pattern E	Pattern E
	5	1,2-Dichloroethane	5:1	Solution	Thin suspension	N/P	No peaks	Pattern A
	5	Butan-1-ol	2:1	Thin suspension	Thin suspension	N/P	Pattern E	Pattern E
	5	Butyronitrile	3:1	Thin suspension	Thin suspension	N/P	Pattern E	Pattern E
	5	MeTHF	1:1	Thin suspension	Thin suspension	N/P	Pattern E	Pattern E
	5	EtOAc	1:1	Thin suspension	Thin suspension	N/P	Pattern E	Pattern E

	5	iPrOAc	1:1	Thin suspension	Thin suspension	N/P	Pattern E	Pattern E
	5	Anisole	5:1	Thin suspension	Thin suspension	N/P	Pattern E	Pattern E
	5	Chlorobenzene	5:1	Thin suspension	Thin suspension	N/P	Consistent with input material (poorly cryst.)	Consistent with input material
	5	Hexafluorobenzene	3:1	Thin suspension	Thin suspension	N/P	Consistent with input material	Consistent with input material

Table S8: Solubility assessment is done using different solvents of amorphous form.

Solvent	Vol of solvent						
	2.5	10	20	30	40	50	50
	25 °C						40 °C
Hexane	N/P	X	X	X	X	X	X
Tetralin	N/P	X	X/+/-	+/-	+/-	+/-	+/-
Acetic acid	N/P	X	X	X	X	X/+/-	X/+/-
Acetophenone	N/P	X/+/-	+/-	+/-	+/-	+/-	+/-
Benzonitrile	N/P	X/+/-	X/+/-	+/-	+/-	+/-	+/-
Benzyl alcohol	N/P	P					
Benzyl alcohol	X/+/-						
1-Butanol	N/P	X	X	X	X	X	X/+/-
Butyronitrile	N/P	X	X	X	X	X	X/+/-
Chlorobenzene	N/P	X	X/+/-	+/-	+/-	+/-	+/-
Chlorobenzotrifluoride	N/P	X	X	X/+/-	X/+/-	+/-	+/-
Chloroform	N/P	X	X	X	X/+/-	X/+/-	+/-
Cyclopentylmethyl ether	N/P	X	X	X	X	X	X
Cyclohexane	N/P	X	X	X	X	X	X
Cyclohexanone	N/P	X	X	X	X	X	X/+/-
1,2-Dichlorobenzene	N/P	X/+/-	+/-	+/-	+/-	+/-	+/-
1,2-Dichloroethane	N/P	X	X	X	X/+/-	+/-	+/-
Dimethoxyethane	N/P	X	X	X	X	X/+/-	X/+/-
Dimethylacetamide	N/P	X	X	X/+/-	+/-	+/-	+/-
Anisole	N/P	X/+/-	X/+/-	+/-	+/-	+/-	+/-
Ethylene glycol	N/P	P					
Ethylene glycol	X						
1-Fluorobenzene	N/P	X	X	X/+/-	X/+/-	+/-	+/-
Glycerol	N/P	X	X	X/+/-	+/-	+/-	+/-
Hexafluorobenzene	N/P	X	X	X	X	X	X

Hexafluoropropan-2-ol	N/P	P					
Hexafluoropropan-2-ol	P						
Methyl-THF	N/P	X	X	X	X	X/+/-	X/+/-
NMP	N/P	X	X/+/-	X/+/-	X/+/-	+/-	P
NMP	X						
Perfluorohexane	N/P	X	X	X	X	X	X
Propionitrile	N/P	X	X	X	X	X	X
Sulfolane	N/P	X	X	X	X/+/-	X/+/-	X/+/-
Trifluoroethanol	N/P	P					
Trifluoroethanol	X						
Trifluorotoluene	N/P	X	X	X	X	X/+/-	X/+/-
o-Xylene	N/P	+/-	+/-	+/-	+/-	+/-	+/-
Cumene	N/P	X	X	X	X	X	+/-
Cyclohexanol	N/P	X	X	X	X	X	+/-
Dimethyl formamide	N/P	X	X	X	X	+/-	+/-
Dimethyl carbonate	N/P	X	X	X	X	X	X
Ethyl formate	N/P	X	X	X	X	X	X
Diisopropyl ether	N/P	X	X	X	X	X	X
Nitrobenzene	N/P	X	X	X	X	+/-	+/-
2-ethoxyethanol	N/P	X	X	X	X	X	X

Table S9: Polymorph screening experiments on samples of Amorphous material post-solubility assessment.

Solvent	After 40 / 25 °C maturation	After 5 °C cool	Evaporation	XRPD 'damp'	XRPD dry
Hexane	Suspension	N/P	N/P	Pattern A + Form 3	Pattern A + Form 3 + 2 peaks
Tetralin	Thin suspension	N/P	N/P	Form 3 + 2 peaks (poorly cryst.)	Form 3+ 2 peaks (poorly cryst.)
Acetic acid	Suspension	N/P	N/P	Form 3 + 2 peaks	Form 3 + 2 peaks
Acetophenone	Thin Suspension	N/P	N/P	Form 3 + some Pattern C	Form 3 + some Pattern C
Benzonitrile	Suspension	N/P	N/P	Form 3	Form 3
Benzyl alcohol	N/P	Solution	Solution	N/P	N/P
Benzyl alcohol	Suspension	N/P	N/P	Pattern D	Pattern D
1-Butanol	Suspension	N/P	N/P	Pattern A	Pattern A
Butyronitrile	Suspension	N/P	N/P	Pattern A	Pattern A
Chlorobenzene	Thin Suspension	N/P	N/P	Form 3	Form 3
Chlorobenzotrifluoride	Suspension	N/P	N/P	Form 3	Form 3
Chloroform	Suspension	N/P	N/P	Form 3	Form 3
Cyclopentylmethyl ether	Suspension	N/P	N/P	Form 3	Form 3
Cyclohexane	Suspension	N/P	N/P	Form 3	Form 3
Cyclohexanone	Suspension	N/P	N/P	Mostly amorphous + Pattern A	Form 3
1,2-Dichlorobenzene	Thin Suspension	N/P	N/P	No peaks	Mostly amorphous
1,2-Dichloroethane	Suspension	N/P	N/P	Pattern A	Pattern A
Dimethoxyethane	Suspension	N/P	N/P	Form 3 + Pattern A	Form 3 + Pattern A + 2 peaks
Dimethylacetamide	Thin Suspension	N/P	N/P	Mostly amorphous	Mostly amorphous
Anisole	Thin Suspension	N/P	N/P	Mostly amorphous	Form 3
Ethylene glycol	N/P	Solution	Solution	N/P	N/P
Ethylene glycol	Suspension	N/P	N/P	Form 3	Form 3 + 2 peaks
1-Fluorobenzene	Suspension	N/P	N/P	Form 3 + Pattern A	Form 3 + 2 peaks
Glycerol	Solution	Solution	Solution	N/P	N/P
Hexafluorobenzene	Suspension	N/P	N/P	Mostly amorphous	Form 3 + 2 peaks

Hexafluoropropan-2-ol	N/P	Brown Solution	N/P	N/P	N/P
Hexafluoropropan-2-ol	N/P	Brown Solution	N/P	N/P	N/P
Methyl-THF	Suspension	N/P	N/P	Form 3 + Pattern A	Form 3 + Pattern C
Hexafluorobenzene	Suspension	N/P	N/P	Mostly amorphous	Form 3 + 2 peaks
NMP	N/P	Solution	Solution	N/P	N/P
NMP	Suspension	N/P	N/P	Form 3	Form 3
Perfluorohexane	Suspension	N/P	N/P	Form 3	Form 3
Propionitrile	Suspension	N/P	N/P	Form 3 + Pattern A	Form 3
Sulfolane	Suspension	N/P	N/P	No peaks	Mostly amorphous
Trifluoroethanol	N/P	Solution	Brown Solution	N/P	N/P
Trifluoroethanol	Suspension	N/P	N/P	Pattern D + Pattern C	Consistent with input material
Trifluorotoluene	Suspension	N/P	N/P	Form 3	Form 3
Cumene	Thin Suspension	N/P	N/P	Form 3	Form 3 + Pattern A
Cyclohexanol	Thin Suspension	N/P	N/P	Mostly amorphous	Mostly amorphous
Dimethyl formamide	Thin Suspension	N/P	N/P	No peaks	Pattern D + 1 peak
Dimethyl carbonate	Suspension	N/P	N/P	Pattern A (poorly cryst.)	Form 3 + Pattern A
Ethyl formate	Suspension	N/P	N/P	Pattern A	Pattern A
Diisopropyl ether	Suspension	N/P	N/P	Form 3	Form 3
Nitrobenzene	Thin Suspension	N/P	N/P	Form 3 (poorly cryst.)	Form 3
2-ethoxyethanol	Suspension	N/P	N/P	Pattern A (poorly cryst.)	Pattern A

Figure S1: (A). NMR of starting material (B). TGA & DSC of starting material

(A)

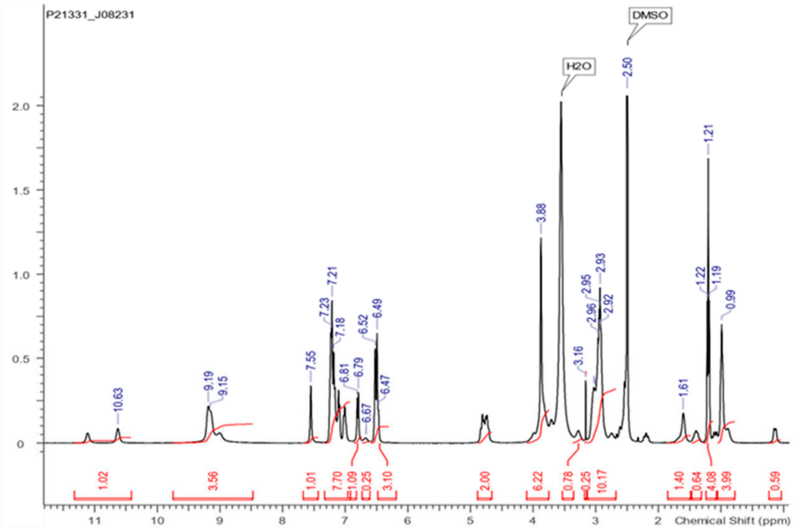

(B)

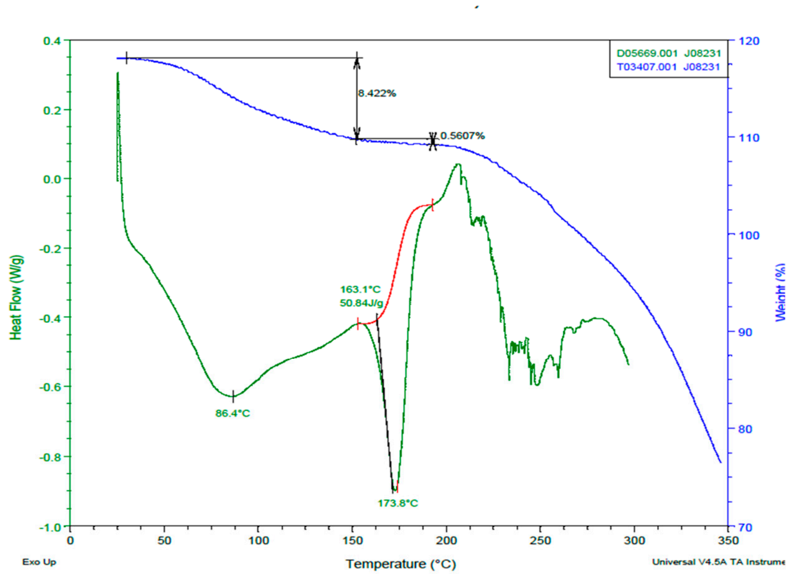

Figure S2: XRPD diffractogram Elacestrant methanol solvate (black) for comparison with experimental XRPD diffractograms of Elacestrant Form 1, Form 2, and Form 3

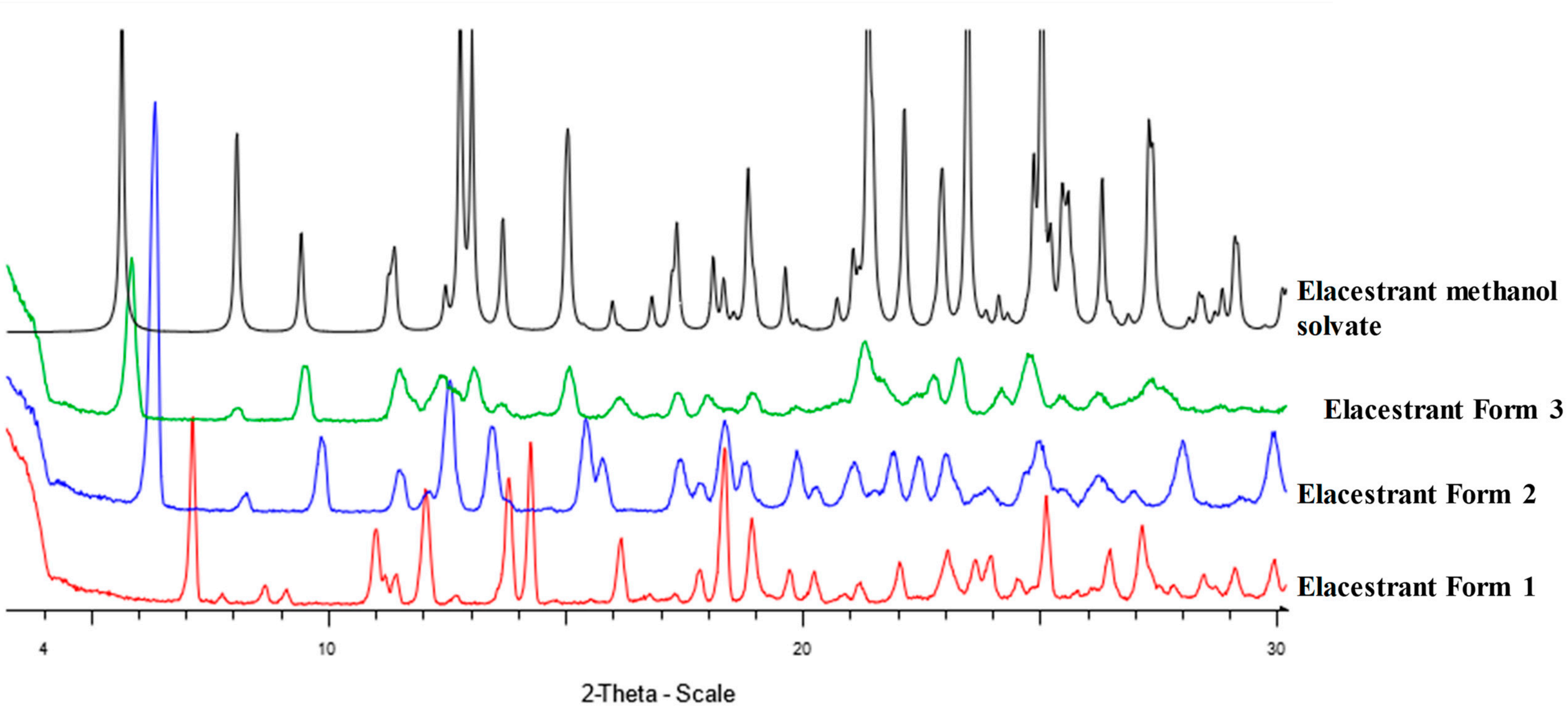

Figure S3: XRPD diffractogram Elacestrant methanol solvate (black) for comparison with experimental XRPD diffractograms of Elacestrant Form 3, Pattern C, and Pattern E

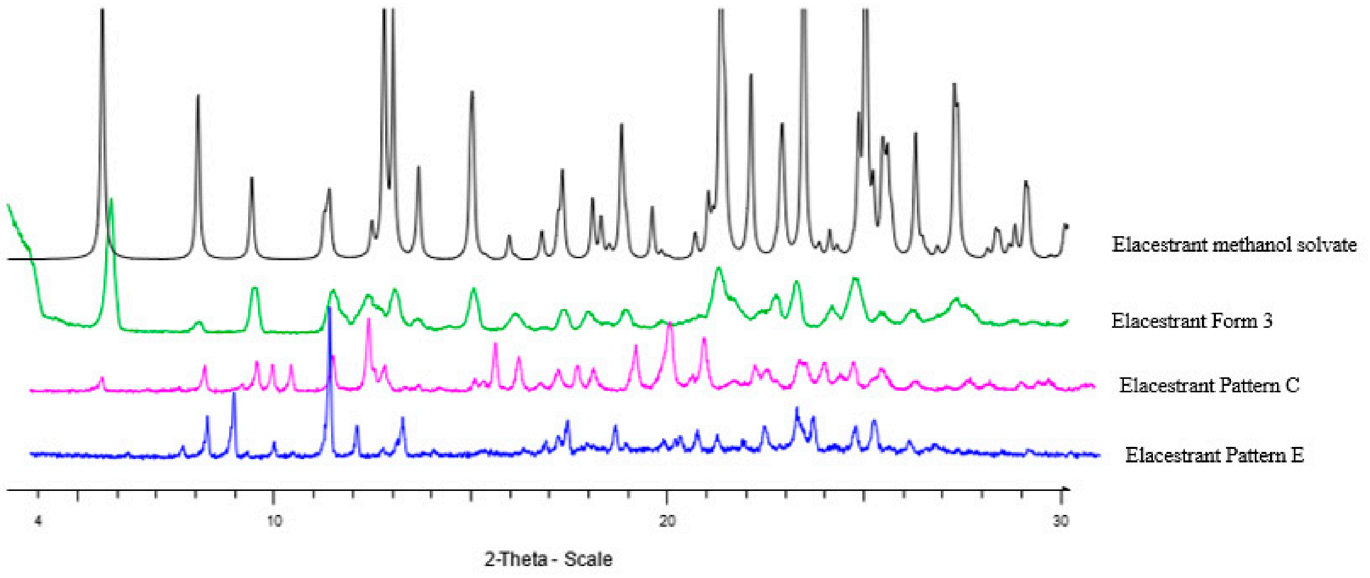

Figure S4: (I) - (IV): XRPD diffractograms of solids obtained after 50 °C / RT maturation or cooling of crystalline material in different solvents. XRPD diffractograms are shown for ‘damp’ and then ‘dry’ samples.

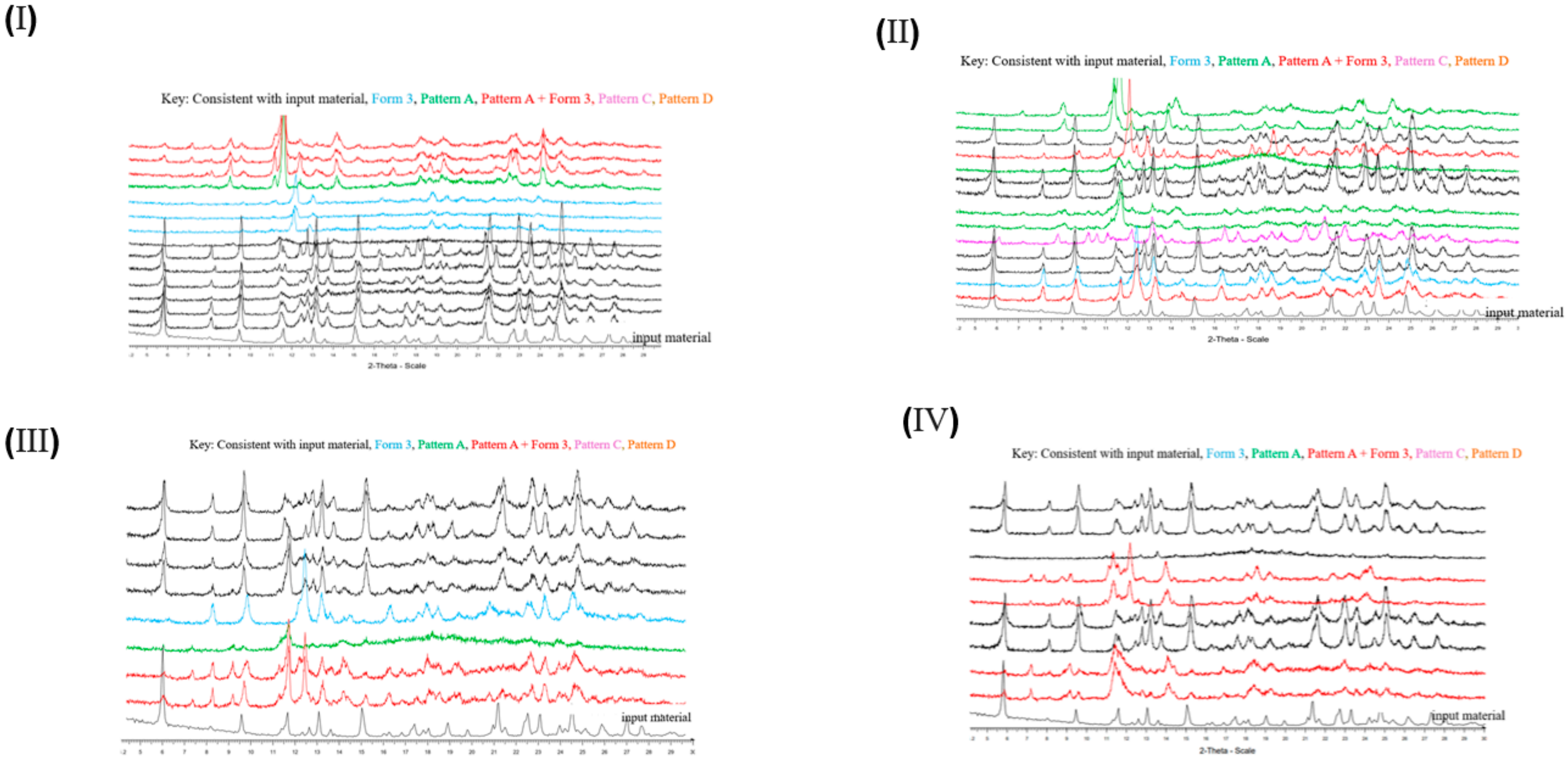

Figure S4: (V)- (VI): XRPD diffractograms of solids obtained after 50 °C / RT maturation or cooling of crystalline material in different solvents. XRPD diffractograms are shown for ‘damp’, then ‘dry’ samples.

(V)

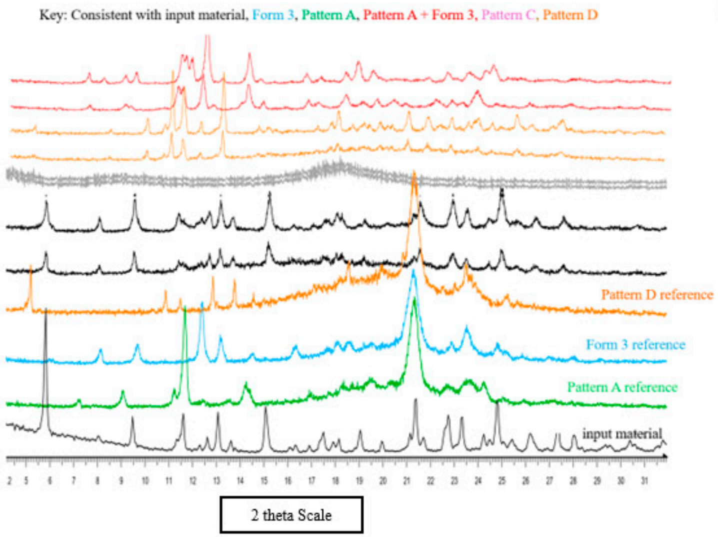

(VI)

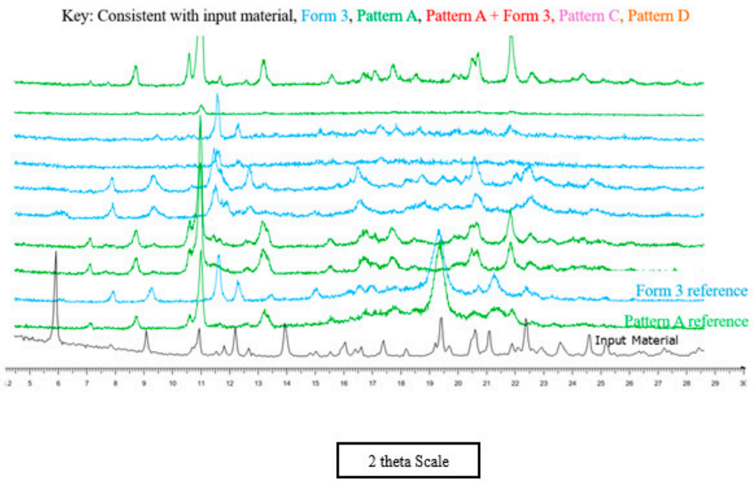

Figure S5: I. XRPD of solids obtained after polymer templating experiments_ EtOH (4% aq.), (II) - (III): XRPD of solids obtained after polymer templating experiments_ MeOH, (IV): XRPD of solids obtained after polymer templating experiments_ Chloroform.

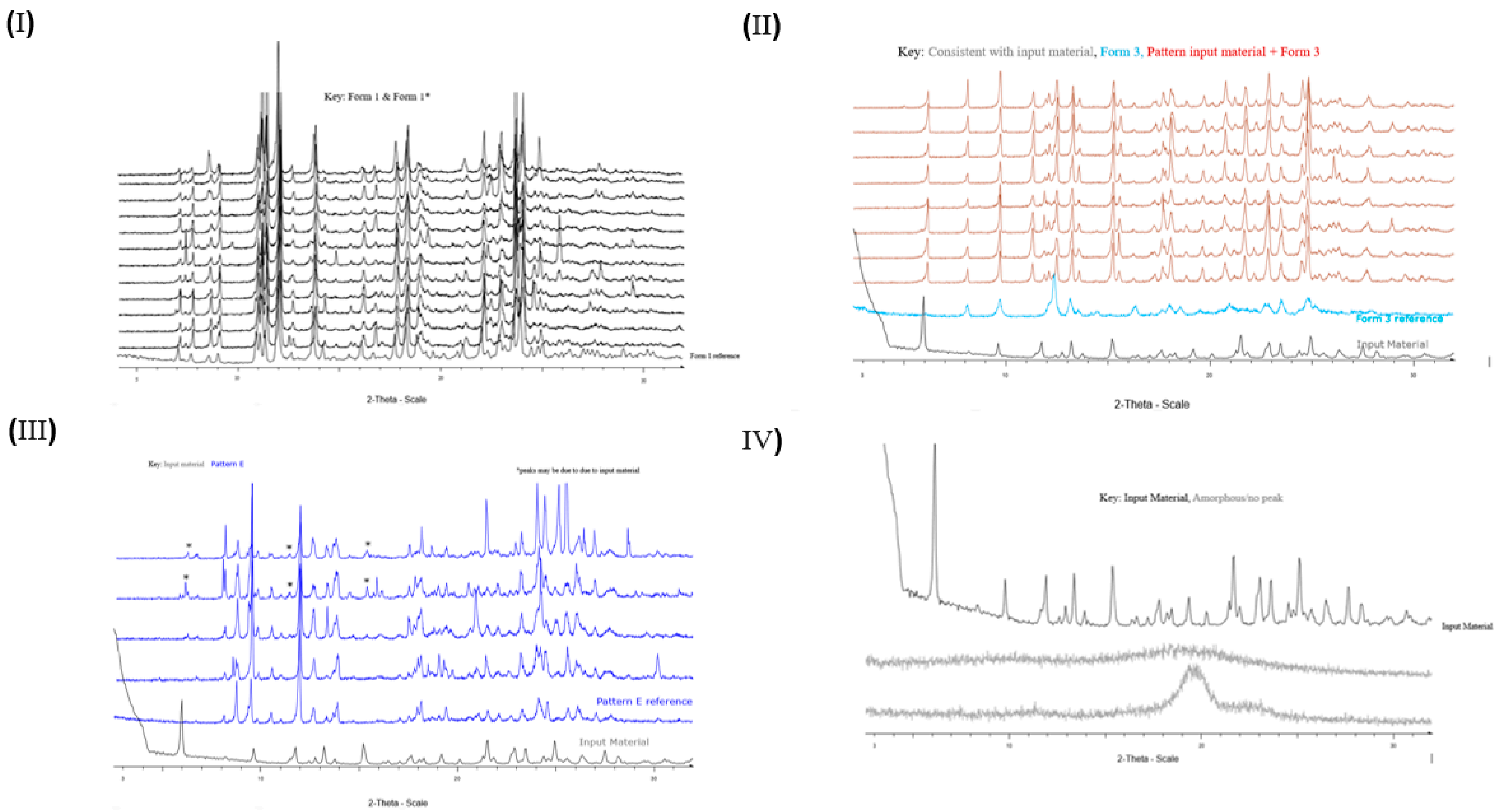

Figure S6: (I) - (IV): XRPD of solids obtained after solvent-drop grinding experiments.

(I)

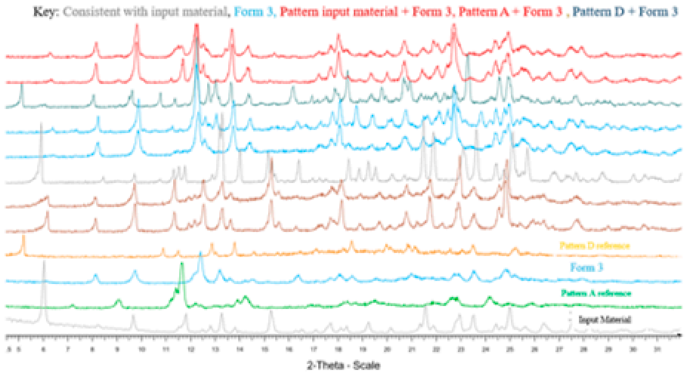

(II)

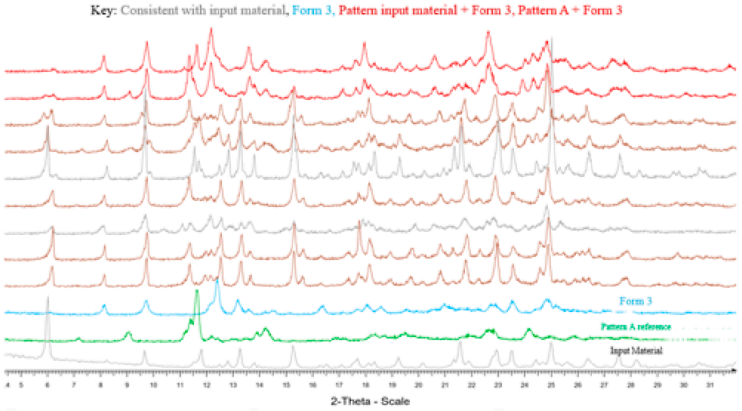

(III)

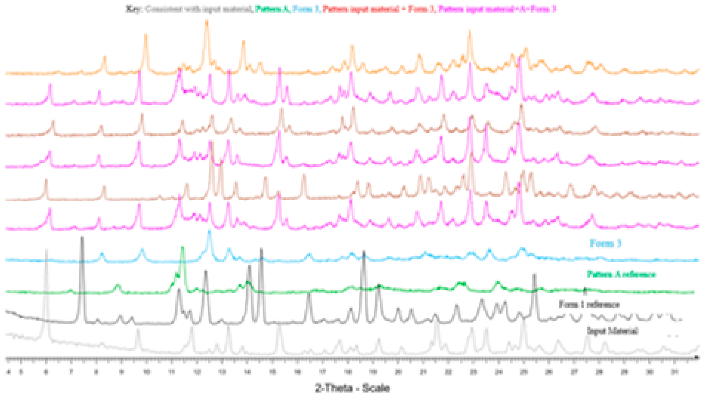

(IV)

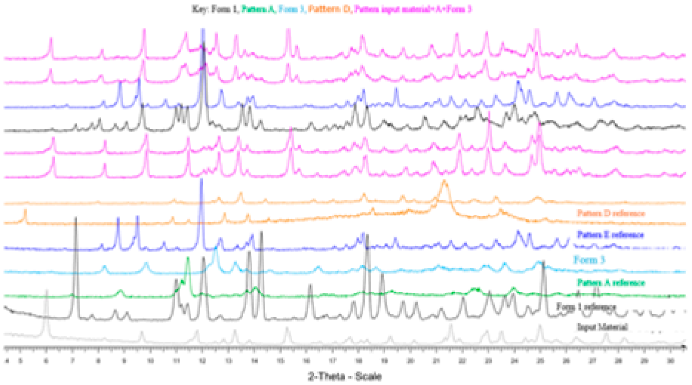

Figure S7: (I): XRPD of solids obtained at 50 °C upon anti-solvent, (II) - (IV): XRPD solids obtained at 5 °C upon anti-solvent are shown damp, then dry.

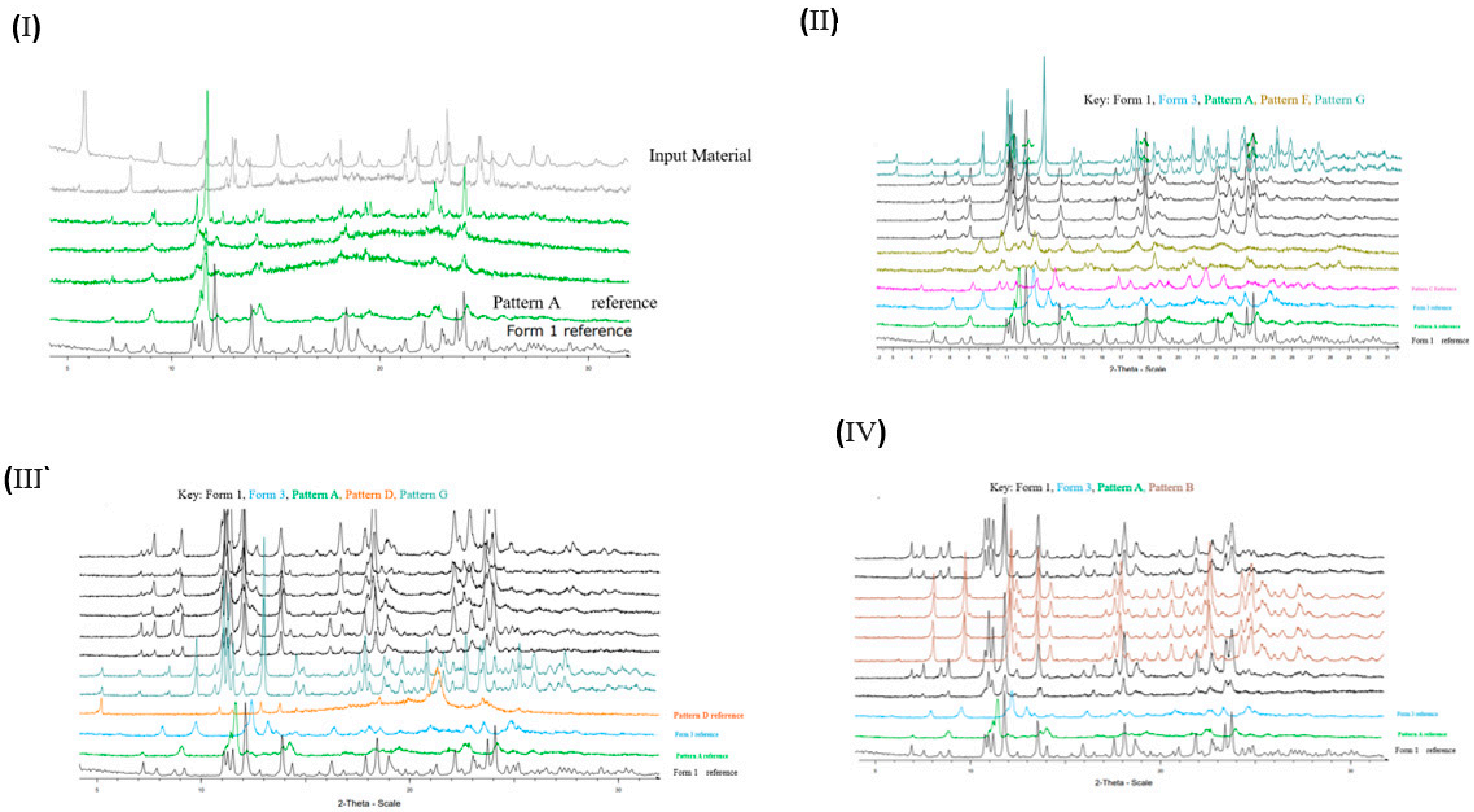

Figure S7: (V-VII): XRPD solids obtained at 5 °C upon anti-solvent are shown damp, then dry.

(V)

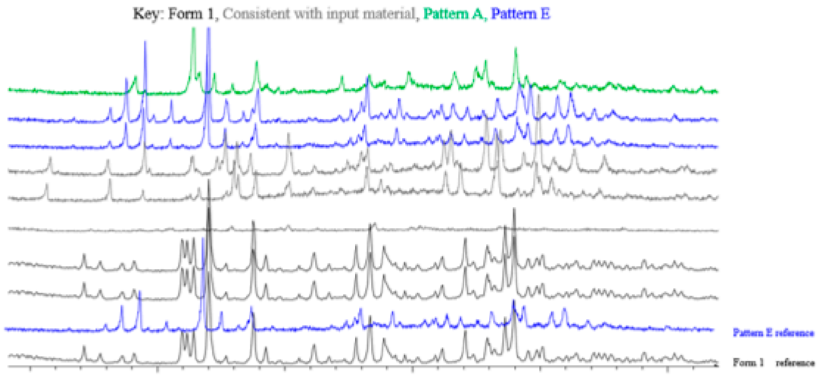

(VI)

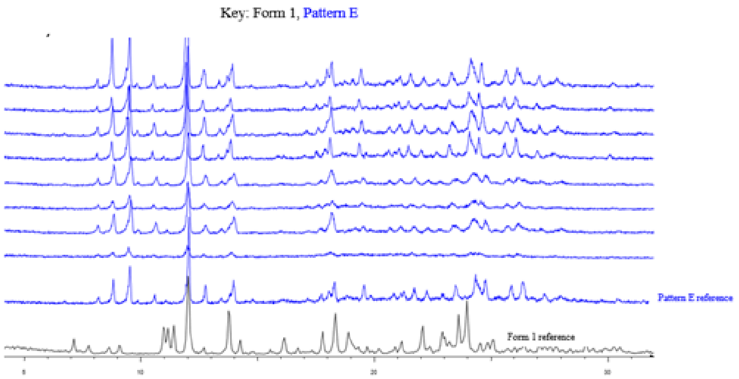

(VII)

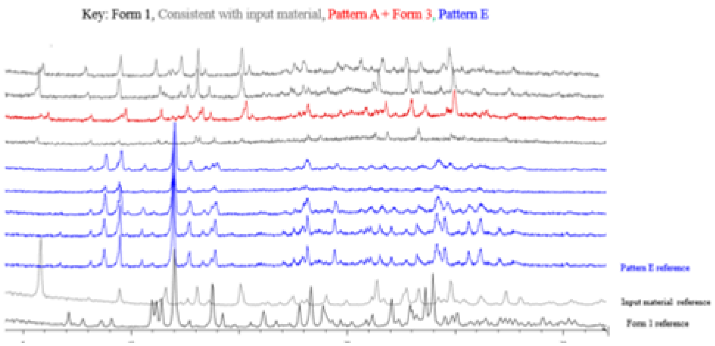

Figure S8: (I) - (IV): XRPD diffractograms of solids obtained after cooling or maturation of Amorphous material in different solvents. XRPD diffractograms are shown for 'damp' and then 'dry' samples.

(I)

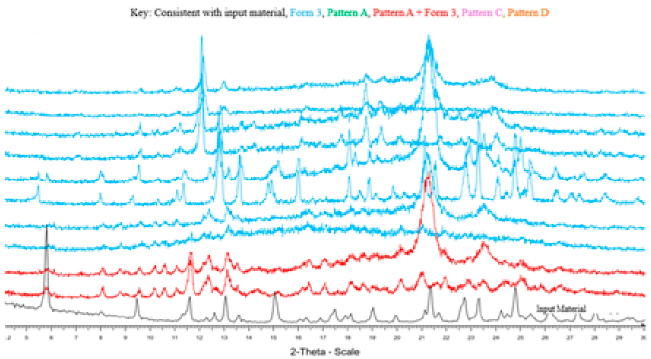

(II)

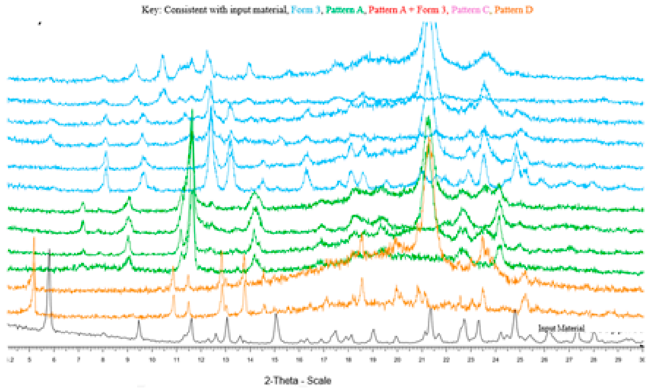

(III)

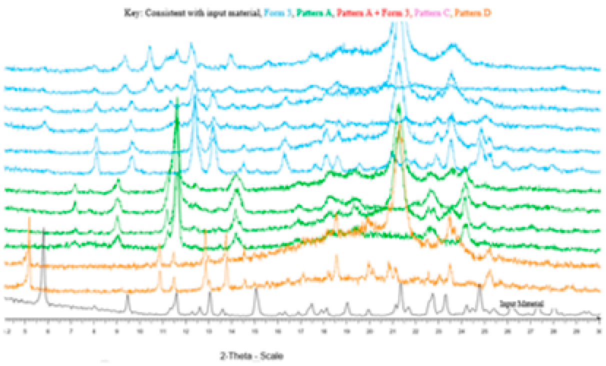

(IV)

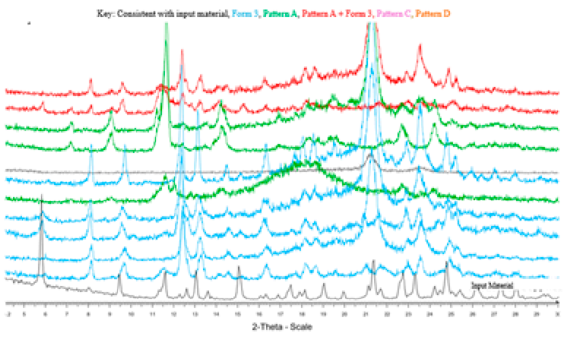

Figure S8: (V)-(VIII): XRPD diffractograms of solids obtained after cooling or maturation of Amorphous material in different solvents. XRPD diffractograms are shown for ‘damp’ and then ‘dry’ samples.

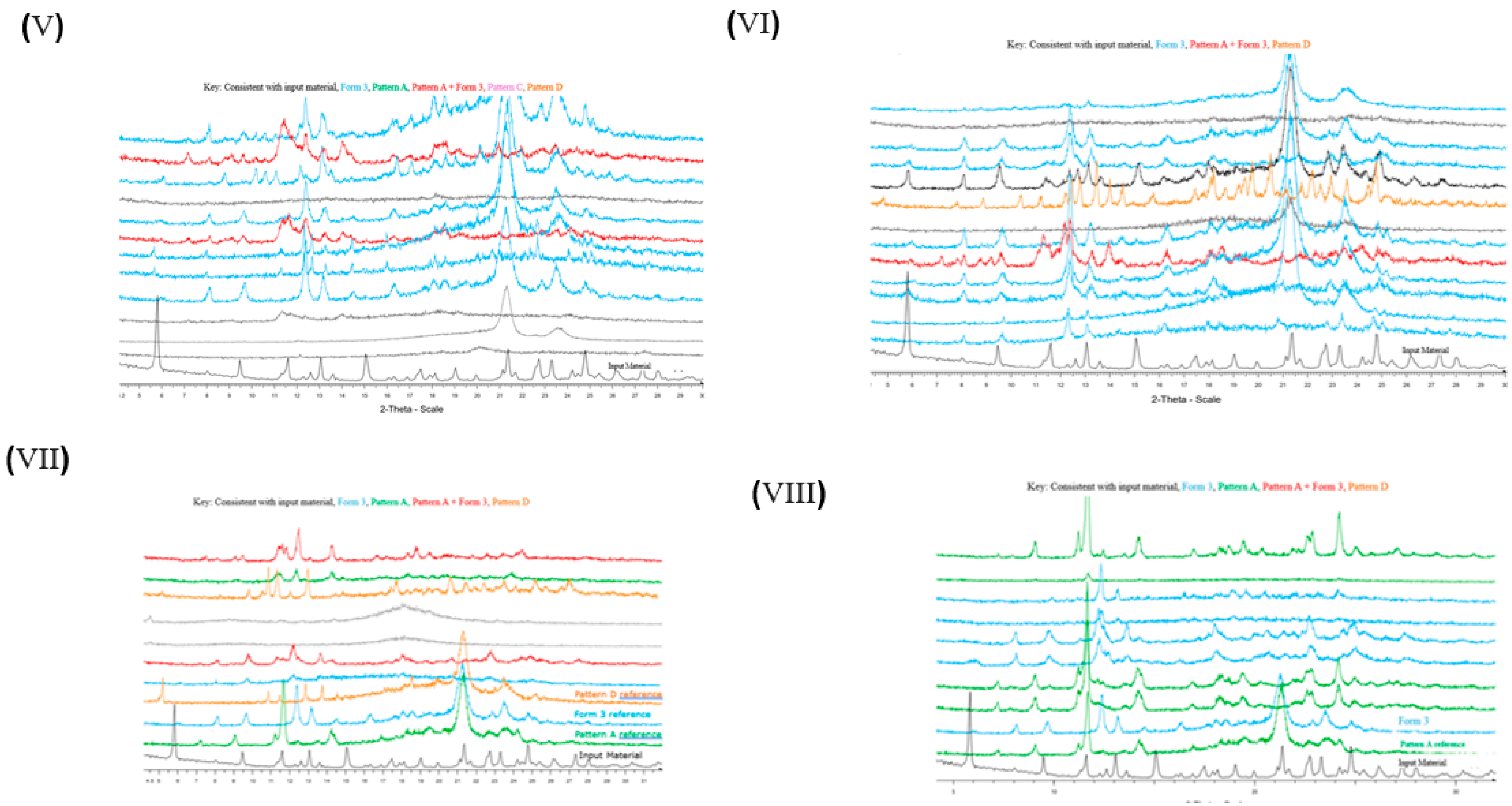

Figure S9: DSC & TGA of the (I) Pattern A, (II) Form B, (III) Form C, (IV) Form 1

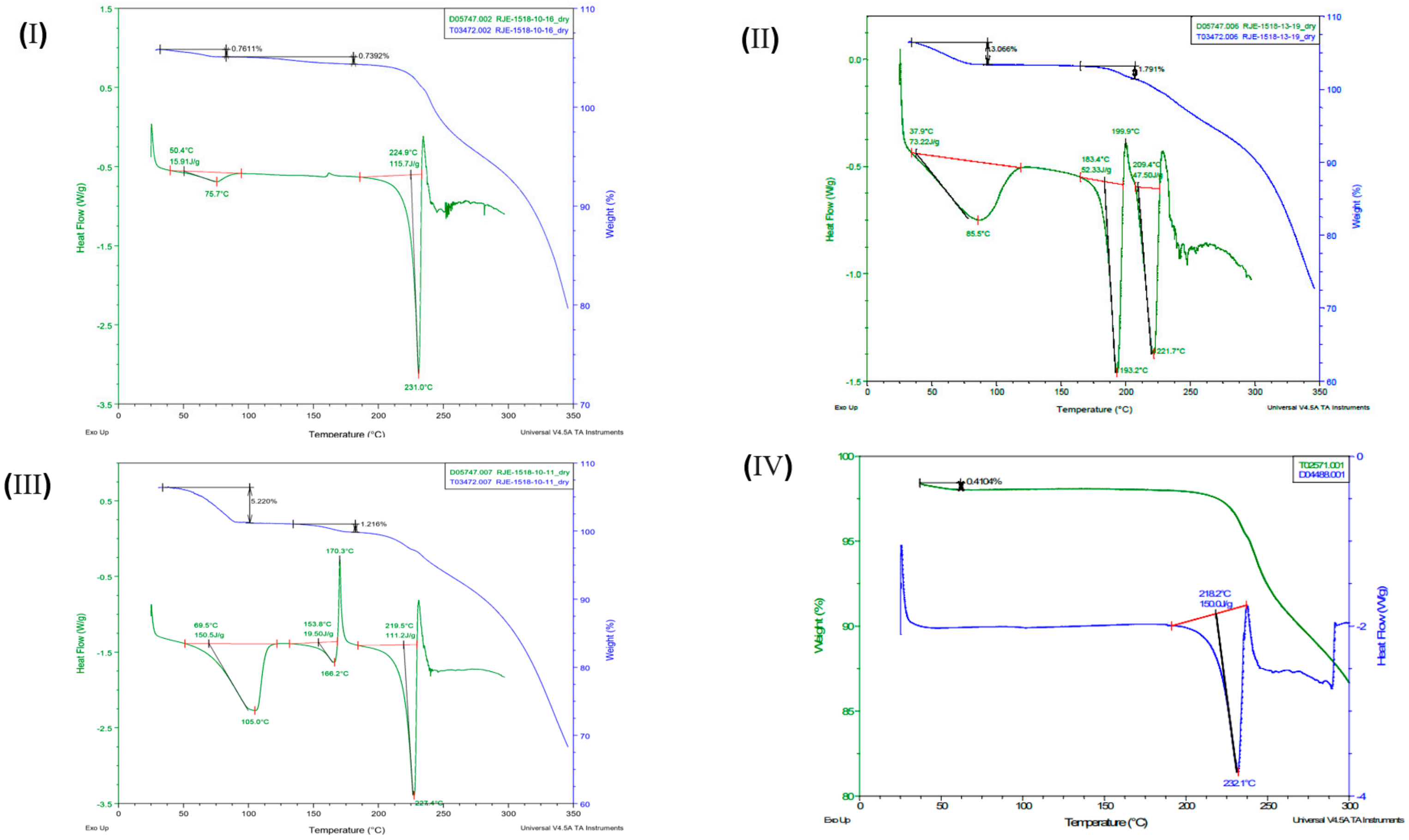

Figure S9: DSC & TGA of the (V) Form E, (VI) Form F, (VII) Form G

(V)

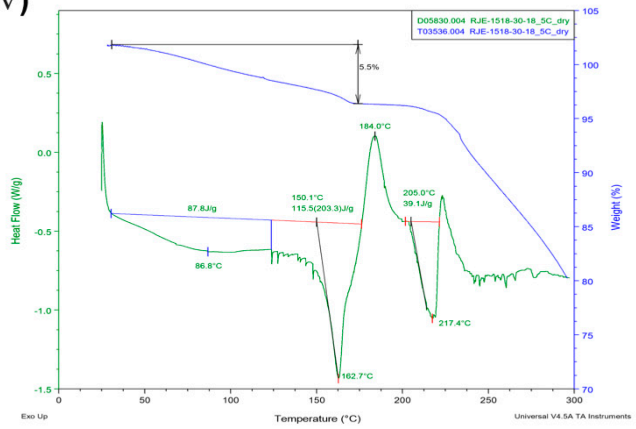

(VI)

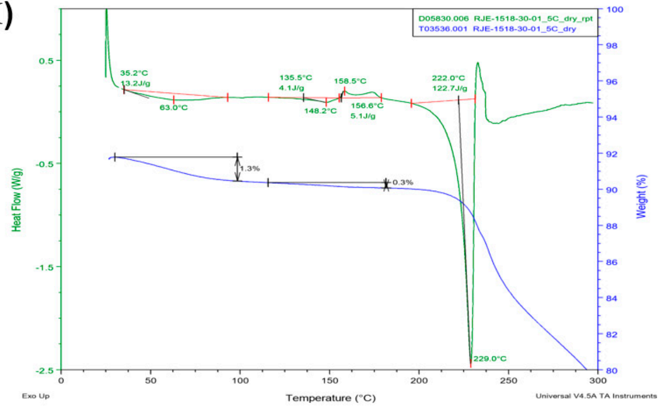

(VII)

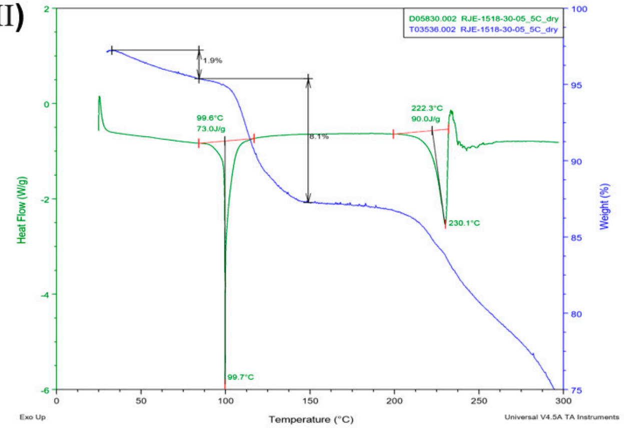

Figure S10: XRPD diffractogram of Pattern A pre-and post-storage at 40 °C/75% RH for 1 week

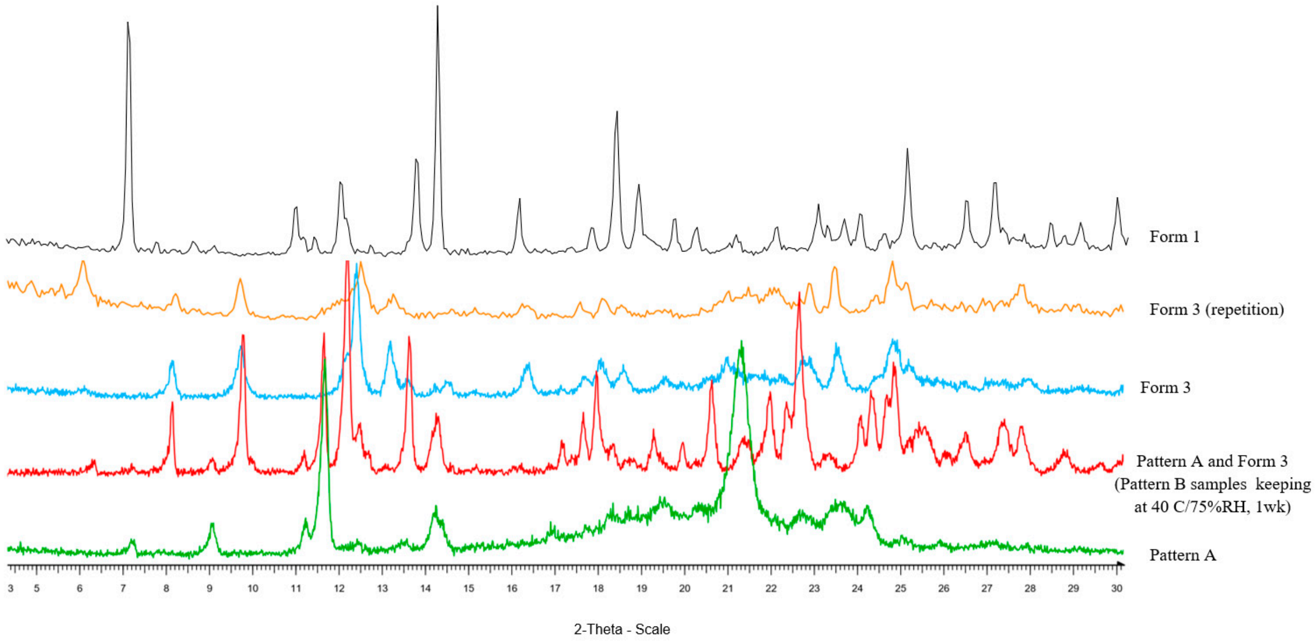

Figure S11: (A) ***Individual XRPD of Form 1 with intensity***

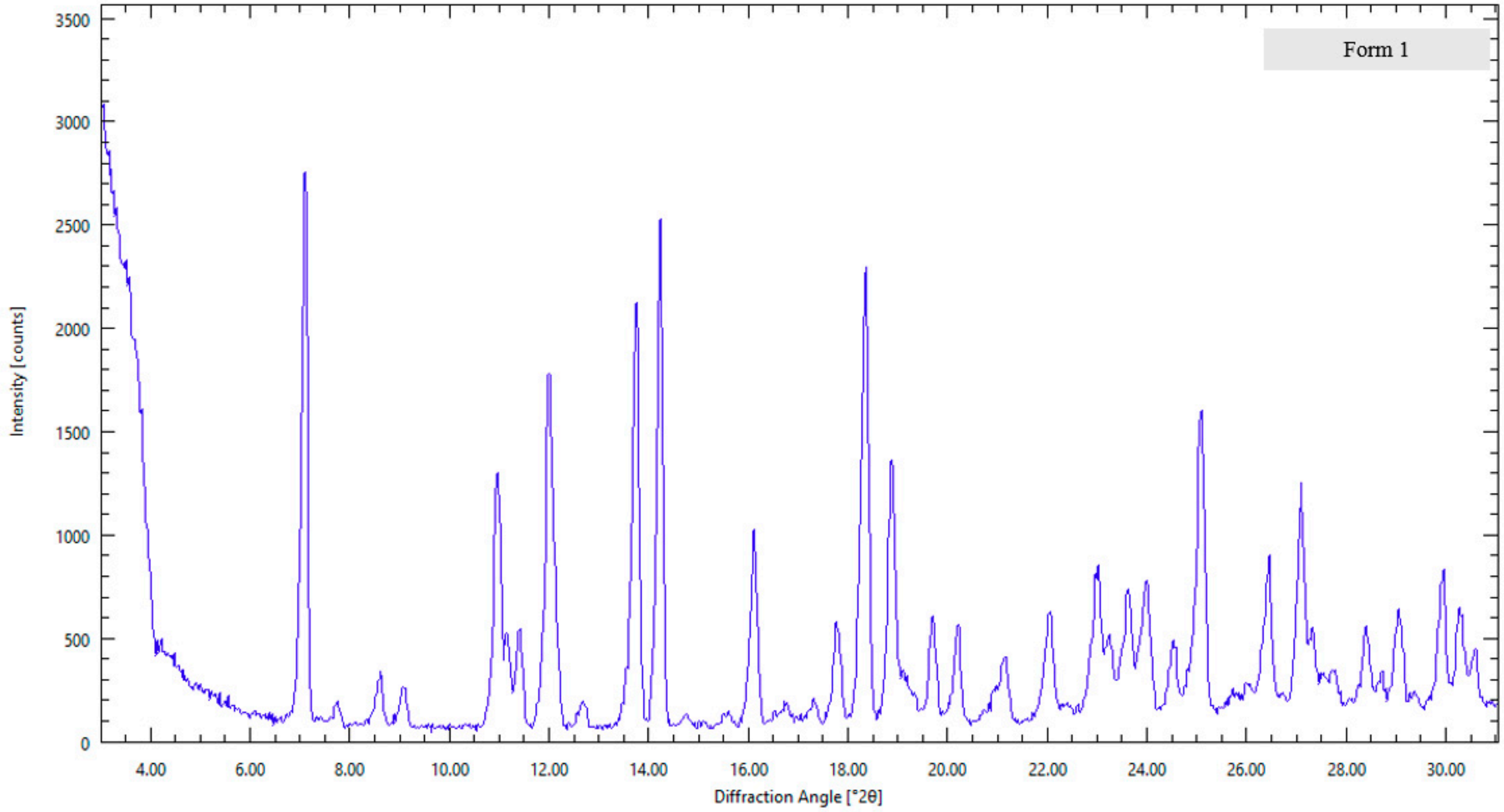

Figure S11: (B) ***Individual XRPD patterns of Input/Starting Materials with intensity***

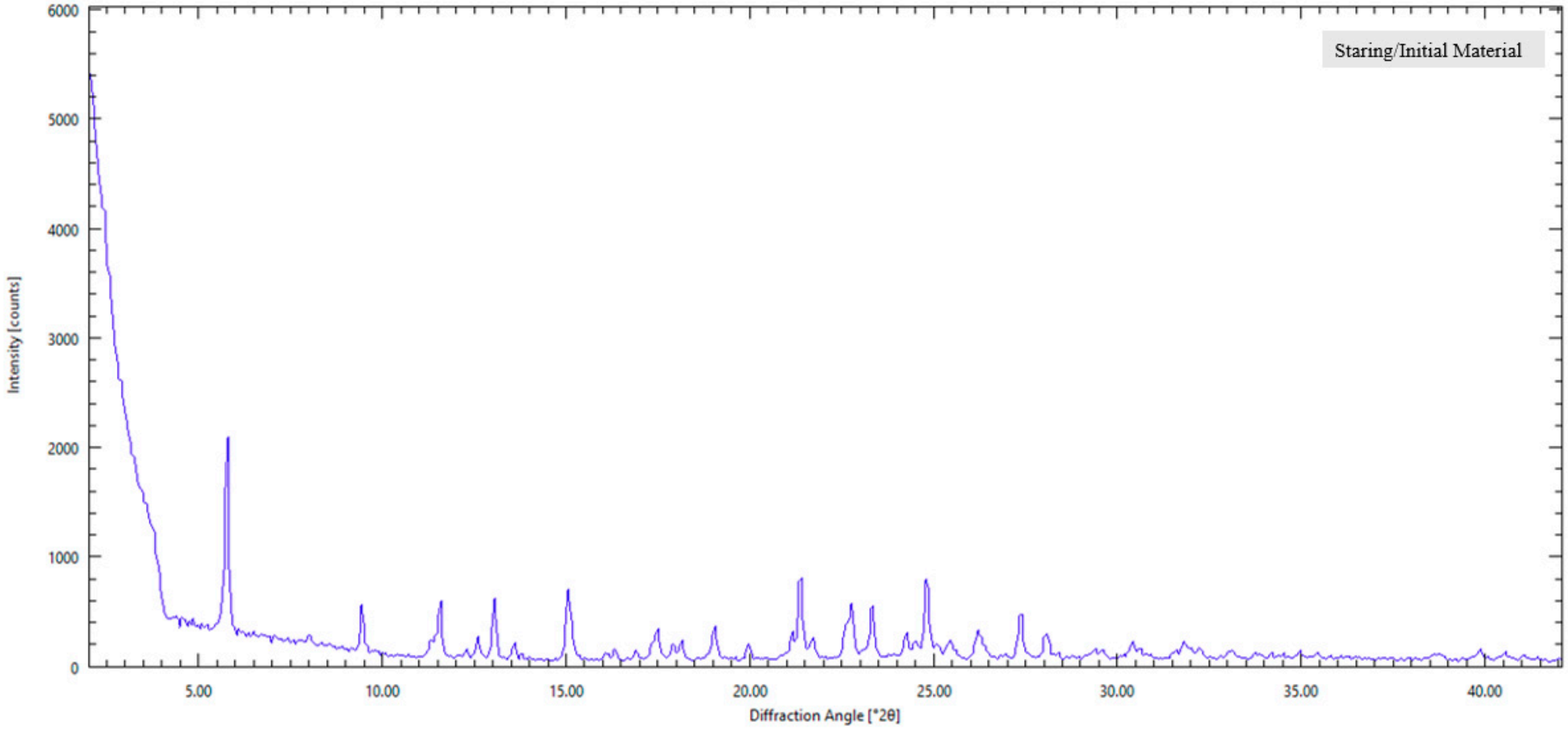

Figure S11: (C) ***Individual XRPD patterns of form 3 with intensity***

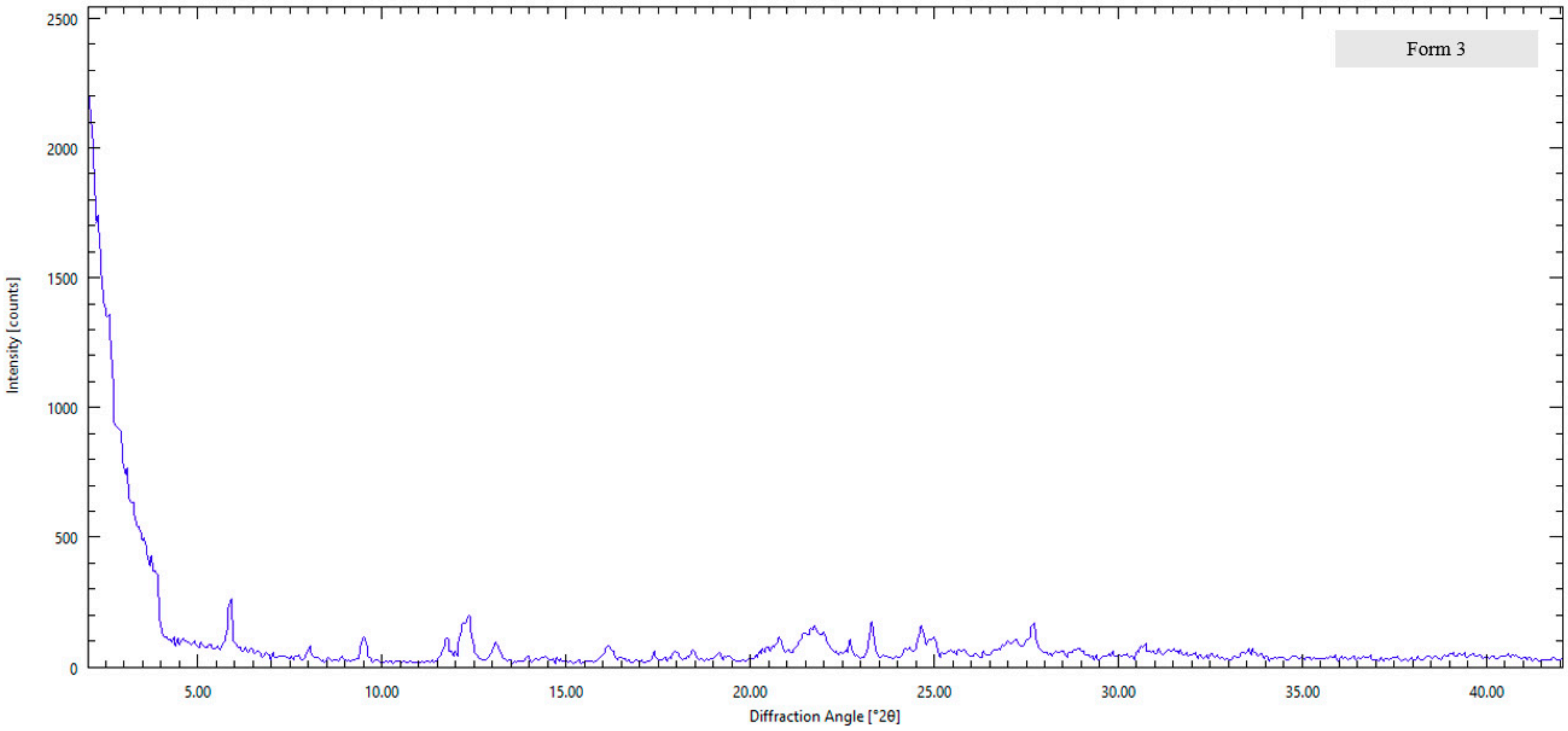

Figure S11: (D) ***Individual XRPD patterns of Pattern A with intensity***

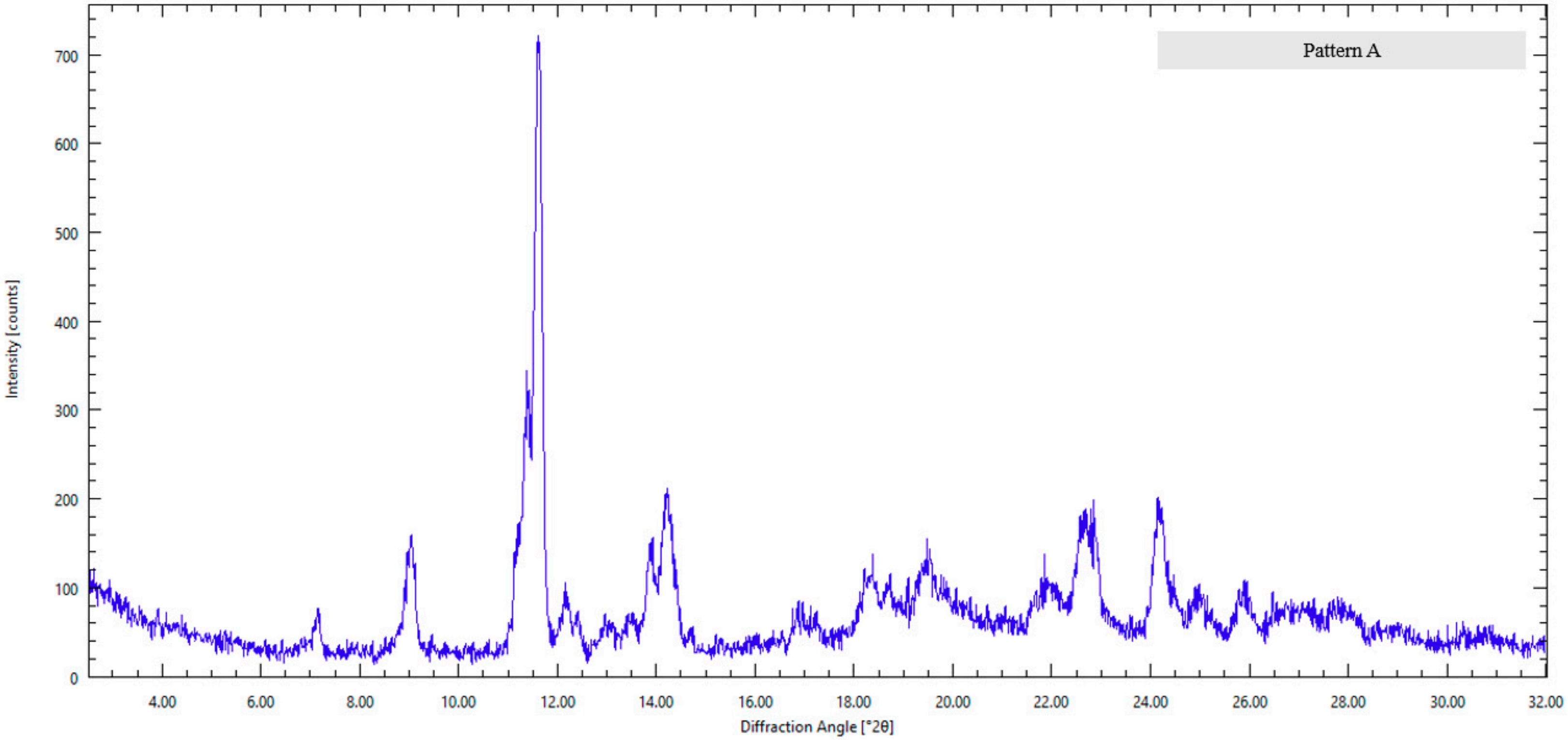

Figure S11: (E) ***Individual XRPD patterns of Pattern B with intensity***

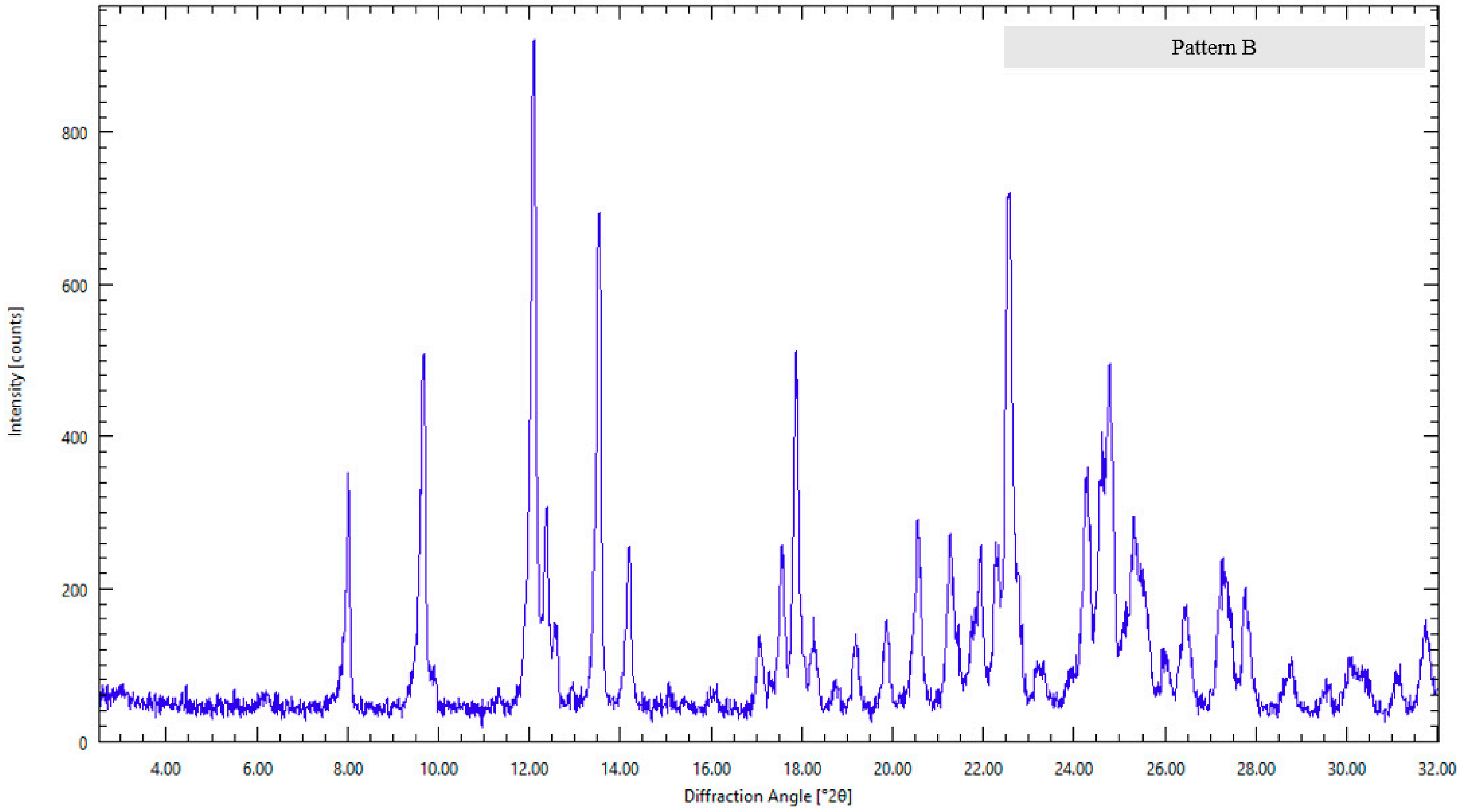

Figure S11: (F) ***Individual XRPD patterns of Pattern C with intensity***

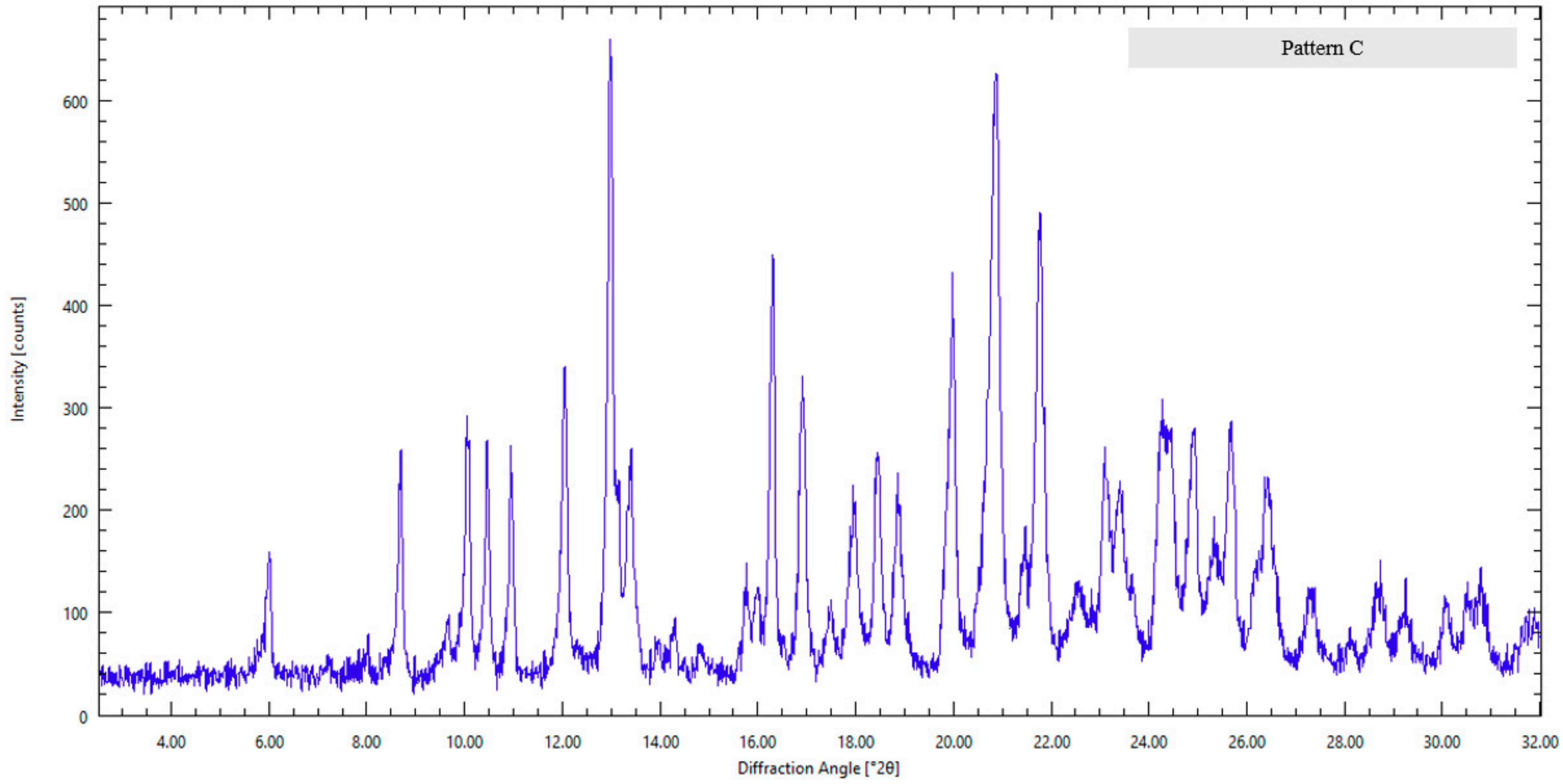

Figure S11: (G) ***Individual XRPD patterns of Pattern D with intensity***

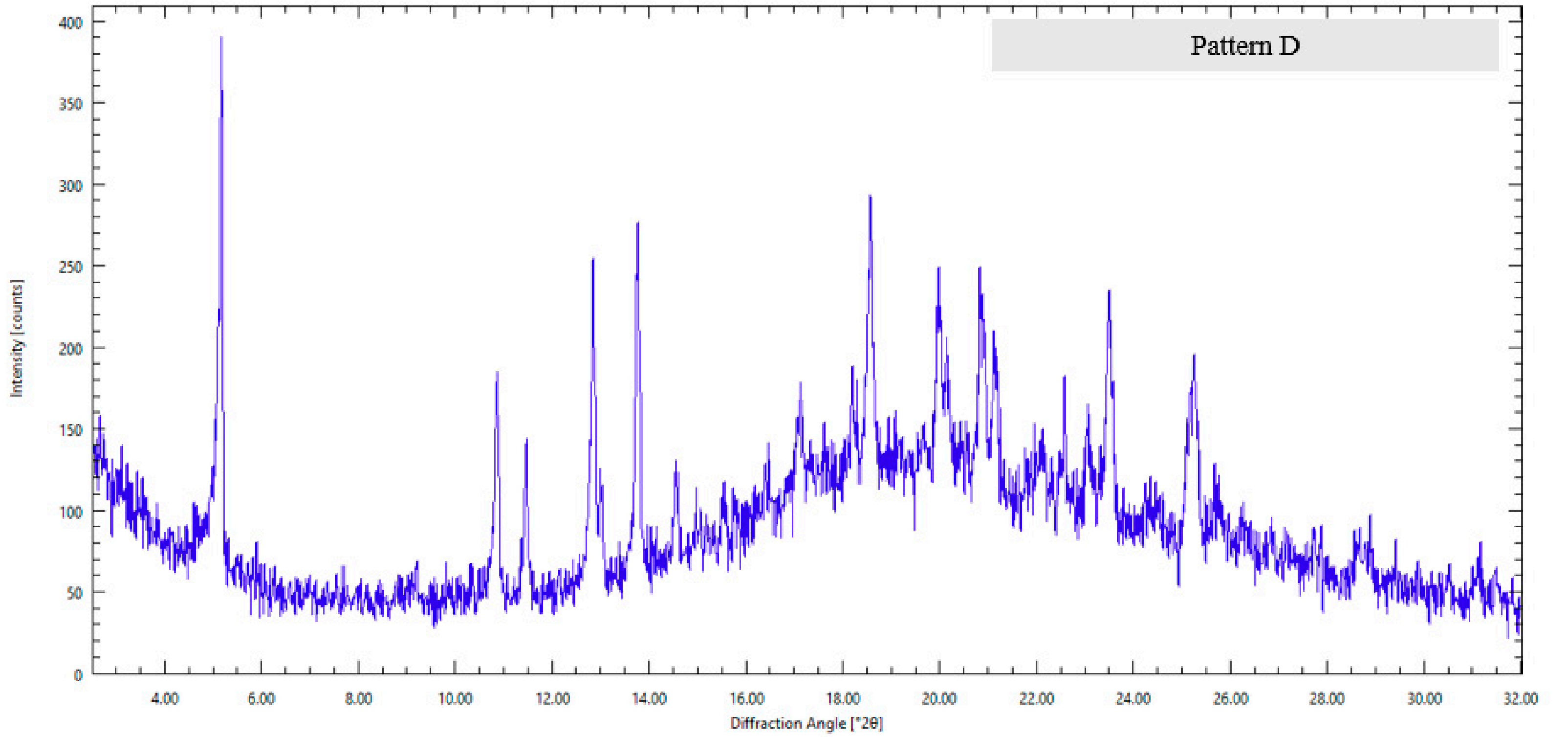

Figure S11: (H) ***Individual XRPD patterns of Pattern E with intensity***

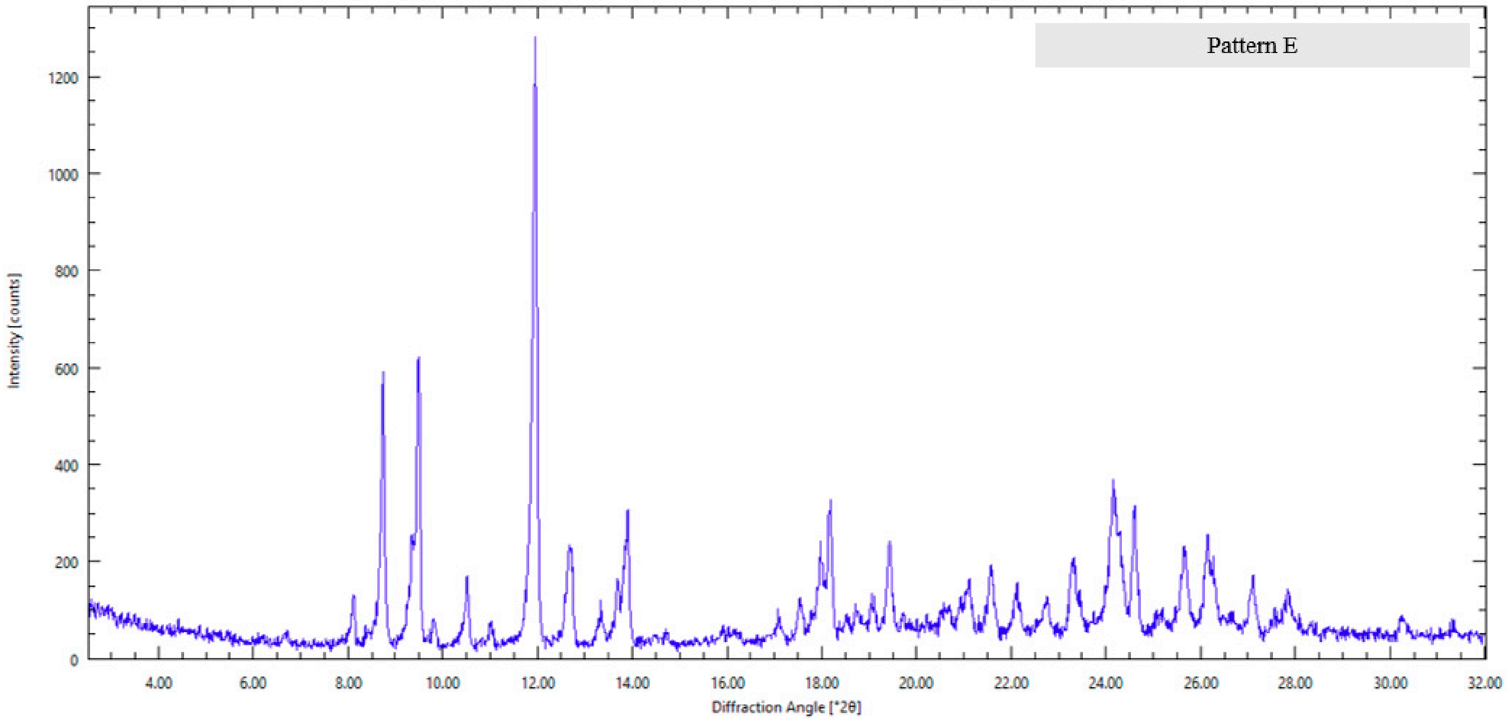

Figure S11: (I) ***Individual XRPD patterns F with intensity***

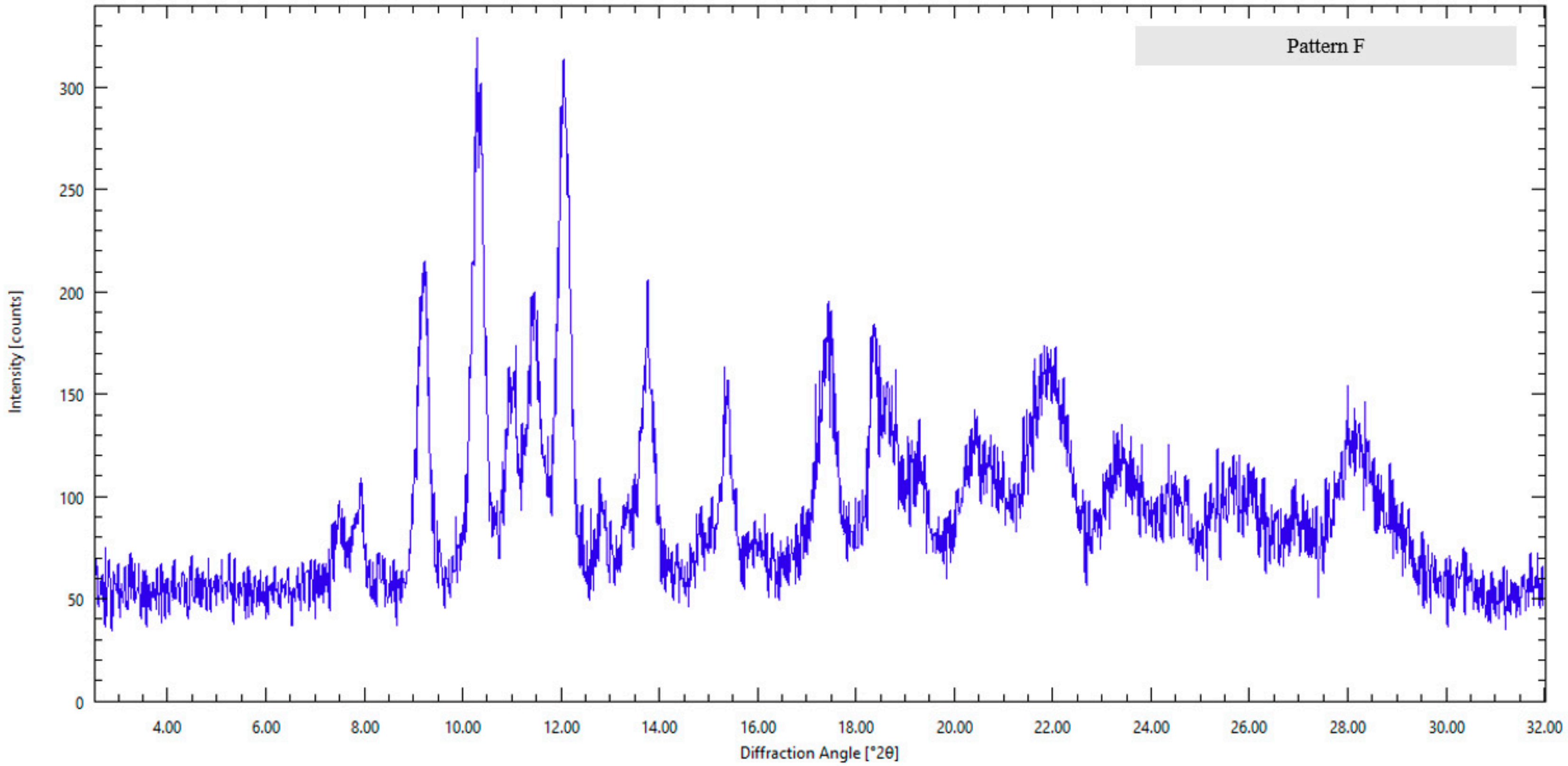

Figure S11: (J) ***Individual XRPD patterns of Pattern G with intensity***

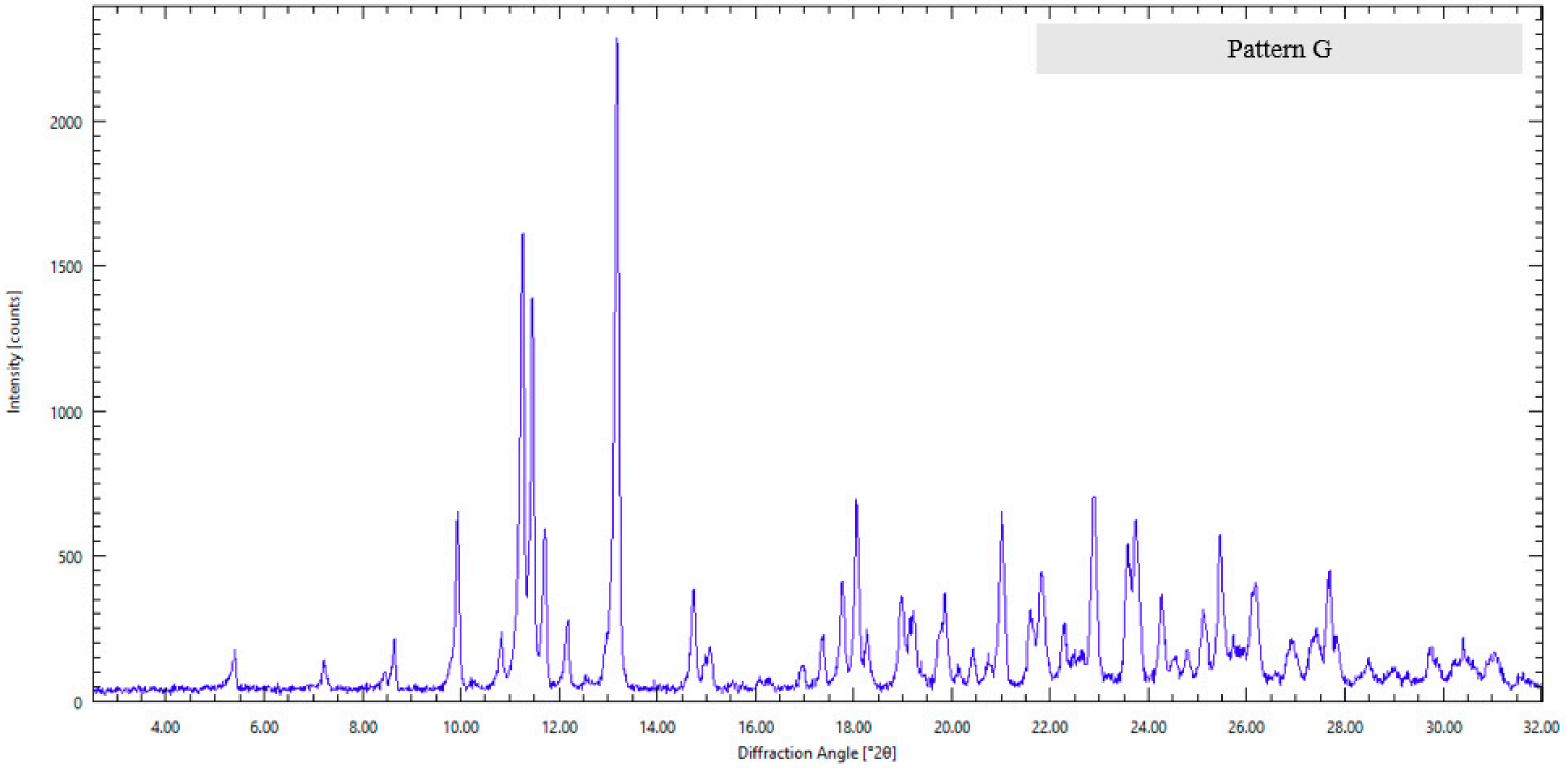

Supplement: Supplementary file 1 [file pharmaceutics-17-00745-s001.zip › pharmaceutics-3578114-supplementary.pdf]
